# Biocompatible Hydrogel for Intra-Articular Implantation Comprising Cationic and Anionic Polymers of Natural Origin: In Vivo Evaluation in a Rabbit Model

**DOI:** 10.3390/polym13244426

**Published:** 2021-12-16

**Authors:** Karina L. Bierbrauer, Roxana V. Alasino, Fernando E. Barclay, Eduardo M. Belotti, Hugo H. Ortega, Dante M. Beltramo

**Affiliations:** 1 Centro de Excelencia en Productos y Procesos de Córdoba, Gobierno de la Provincia de Córdoba, Pabellón CEPROCOR, Santa Maria de Punilla, Córdoba CP 5164, Argentina; bierbrauerk@yahoo.com.ar (K.L.B.); v_alasino@yahoo.com.ar (R.V.A.); 2 Consejo Nacional de Investigaciones Científicas y Técnicas, CONICET, Godoy Cruz 2290, Buenos Aires C1425FQB, Argentina; belottiem@hotmail.com.ar (E.M.B.); hhortega@fcv.unl.edu.ar (H.H.O.); 3 Instituto Argentino de Diagnóstico y Tratamiento SA (IADT) en Ortopedia y Traumatología, Cirugía Artroscópica y Medicina del Deporte, Marcelo T. de Alvear 2346/2400, Buenos Aires C1122AAL, Argentina; fernandoebarclay@gmail.com; 4 Centro de Medicina Comparada, Instituto de Ciencias Veterinarias del Litoral (ICiVet-Litoral), Universidad Nacional del Litoral (UNL), Esperanza 3080, Argentina; 5 Facultad de Ciencias Veterinarias del Litoral, Universidad Nacional del Litoral (UNL), Esperanza 3080, Argentina

**Keywords:** polyquaternium-10, chondroitin sulfate, hydrogel, osteoarthritis, intra-articular implant, rabbit model

## Abstract

We describe the functional capability of a cross-linked hydrogel composed of sulfated glycosaminoglycans and a cationic cellulose by conducting trials on experimental animal models using intra-articular implants to treat an articular disease called osteoarthritis. Forty-eight mature New Zealand white rabbits were divided into three experimental groups: A, B, and C. Group A and B underwent unilateral anterior cruciate ligament transection (ACLT) of the right knee. Subsequently, both knees of group A were treated with the injectable formulation under study. Meanwhile, group B was treated with sterile PBS (placebo). The animals of group C were surgically operated in both knees: Commercial hyaluronic acid (HA) was implanted in the left knee, and the formulation under study was implanted in the right knee. After implantation, all specimens underwent several evaluations at 3, 6, and 12 months postoperatively. At 6 months, no significant differences were detected between the right and left knees of the different groups. However, significant differences were observed between both knees at 12 months in group C, with less cartilage damage in the right knees implanted with our hydrogel. Therefore, in vivo studies have demonstrated hydrogel safety, superior permanence, and less cartilage damage for long-term follow up 12 months after implantation for the formulation under study compared with commercial HA.

## 1. Introduction

Osteoarthritis (OA) is a disease that involves entire articulations, which is usually prone to structural damage due to changes that occur in synovial liquid properties. There is no definitive cure for OA, its pathogenesis is not entirely clear, and the best strategy to deal with OA is prevention [1]. The recommended management of the symptomatology of knee OA consists of conservative, non-pharmacological strategies in the first instance, then the use of pharmacological therapies, and, as a last resort, surgical options [1,2,3]. The injection of viscosupplementation substances is an option prior to surgery [4]. Regarding viscosupplementation, the main compound used currently is hyaluronic acid (HA), which is presented in different formulations [5,6].

Cross-linked HA has shown several advantages: It allows for longer activity within the body and has greater viscoelastic properties [7,8]. Raman et al. [9] implanted high-molecular-weight HA (Hylan G-F20) in the treatment of knee osteoarthritis and demonstrated a reduction in pain for 12 months, but several localized reactions have been observed for HA knee injections in many patients [10]. As an alternative to what is currently available in the market, we developed and evaluated a hydrogel comprising a combination of sulfated glycosaminoglycans with cationic polymers, chondroitin sulfate, and Polyquaternium-10 (PQ10), which have better stability properties. To the best of our knowledge, the PQ10 polymer has only been previously studied [11,12] for its cosmetic uses and has no reference for other types of application.

These polymers were chosen because chondroitin sulfate (CS), a natural biopolymer composed of a chain of alternating sugars (N-acetylgalactosamine and glucuronic acid), is one of the most important structural components of cartilage [13]. CS loss in cartilage is one of the causes of osteoarthritis disease [14,15]. Second, PQ10 is a hydroxyethyl cellulose substituted with quaternary amine groups that has high solubility in aqueous media, bioadhesivity, and mucoadhesion and what are probably the two most important properties, biocompatibility [16] and biostability, because it does not experience enzymatic degradation by eukaryotic cells [17,18,19,20]. The aim of this combination is to obtain a bioadhesive, i.e., biocompatible hydrogel, with rheological properties that allow for longer activity times at the application site without adverse effects. These properties were improved as the presence of cationic groups provides a high affinity for oppositely charged molecules, thus generating materials with rheological properties that are considerably different from the starting polymers [20,21,22,23,24].

One of the chemical approaches for combining PQ10 with CS to improve stability is to cross link the polysaccharide chain to form a molecular network. In this study, we used divinylsulfone (DVS) as an inter-crossing agent [6], which provided the formation of a transparent, elastic, and mucoadhesive hydrogel without influencing the ionic groups of both polymers. This chemical combination resulted in a material that is useful in biomedical applications such as intra-articular implants.

In conclusion, this study describes an “in vivo” study for comparing the effect of a single dose of high-molecular-weight HA (Hylan G-F20–Synvisc one–Genzyme Biosurgery) and our hydrogel PQ10-CS-DVS to evaluate the performance and biological safety of an intra-articular implant in a rabbit model with surgically induced OA.

## 2. Materials and Methods

High-viscosity cationic hydroxyethylcellulose, Polyquaternium-10 (PQ10), also known as SensomerTM 10M was provided by Lubrizol Advanced Materials, INC. (Cleveland, OH, USA). The viscosity of PQ10 solution 1% (*w*/*v*), measured by Brookfield viscometer and cone-plate geometry (CP52) at 20 °C, was between 778 and 2083 cp in the speed range 10–0.5 RPM, respectively. Chondroitin sulfate (CS) sodium salt was provided by Laboratory Syntex S.A. (Buenos Aires, Argentina) and was used as received. The determination of CS molecular weight (MW) was performed using the SDS-PAGE technique with polyacrylamide gel electrophoresis. The molecular weights obtained from this procedure ranged between 45,000 and 66,000 Daltons. Glycine and divinylsulfone (DVS) were obtained from Sigma Aldrich (St. Louis, MO, USA). Figure S1 (Supplementary Materials) shows the molecular structure of PQ10, CS, and DVS. Phosphate-buffered saline solution (PBS), sodium hydroxide, and chloridric Acid (38%) were provided by Biopack (Buenos Aires, Argentina). Water purified by reverse osmosis (MilliQ, Millipore, Madrid, Spain) with a resistivity of >1.82 MΩ cm was used.

### 2.1. Preparation of the Crosslinked PQ10-CS-DVS

**Scheme 1:**
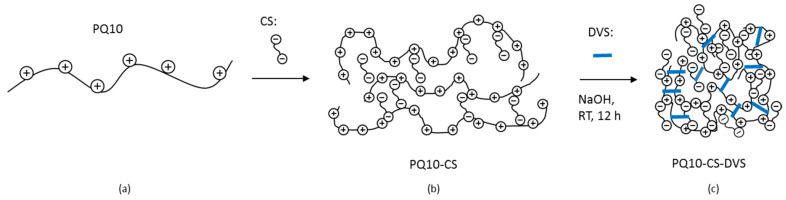
Chemical processes involved in the formation of PQ10-CS-DVS hydrogel. (**a**) Cationic cellulose chains (PQ10) and polyanion (CS) in aqueous solution. (**b**) Electrostatic interaction between PQ10 and CS. (**c**) Hydrogel obtained by crosslinking with DVS.

PQ10 and CS were solubilized in distilled water; PQ10 was prepared in a concentration of 2–4% (*w*/*v*) and CS in a 10% (*w*/*w*) in relation to PQ10 in all cases, since this proportion is the one that presented the maximum viscosity in our previous work [20]. Then, each solution was mixed under vigorous stirring at room temperature for 5 min to allow for electrostatic interaction between the two polymers. The mixture was then brought to pH 12 with 50 mM NaOH final concentration. Afterwards, DVS, previously dissolved in distilled water, was added in proportions ranging from 1 to 5% of the initial mass of the two polymers combined. The entire procedure was carried out at room temperature under continuous stirring. The hydrogel formed was permitted to stand for at least 12 h to complete the reaction. Scheme 1 shows the general procedure. Then, the hydrogel was neutralized by the addition of HCl 50 mM for one hour. The supernatant was removed, and the hydrogel was successively washed with PBS buffer with 0.1% *w/v* of glycine to eliminate the remaining crosslinking agent and to bring the pH of the hydrogel to physiological conditions. Washing cycles were carried out for at least one week. The final pH of the hydrogel supernatant, obtained using a Lutron Model 208 pH meter at 25 ± 2 °C, was between 6.5 and 7. The hydrogel was kept between 4 and 8 °C until sterilization. Table S1 (Supplementary Materials) summarizes the different ratios of PQ10-CS-DVS used to produce the hydrogels. The sterilization method used was an autoclave cycle at 1 atm for 15–20 minutes in all cases.

### 2.2. Hydrogel Characterization

#### 2.2.1. FTIR Measurements

IR spectra were recorded at ambient temperature on a Shimadzu^®^ FTIR spectrometer (Tokyo, Japan) using an accumulation of 16 runs in each sample in the diffuse-reflectance mode.

#### 2.2.2. Differential Scanning Calorimetric (DSC) Analysis

Calorimetric thermal analysis was performed using a Perkin Elmer^®^ Pyris 1 calorimeter (Waltham, MA, USA). The samples were subjected to a nitrogen atmosphere using a standard Indium calibration and an aluminum capsule material. The samples were then sent to a first heating cycle from an initial temperature of −35 °C to a final temperature of 200 °C and then to a reverse cooling cycle, both at a rate of 10 °C per minute. After that, the samples were subjected to a second heating cycle under the same conditions.

#### 2.2.3. Thermogravimetric (TGA) Analysis

Thermogravimetric analysis (TGA) was carried out using a Shimadzu^®^ TGA-50 Thermogravimetric analyzer (Tokyo, Japan). Samples were heated at a rate of 10 °C per minute from a starting temperature of 23 °C to a final temperature of 600 °C in an atmosphere of nitrogen 5.0, using nickel standard (Curie temperature) calibration.

#### 2.2.4. Texture Analysis

We used a TAXT2 texturometer (Stable Micro Systems, Surrey, UK) equipped with a 25 kg load cell to determine the extrusion pattern as a measure of the force required to drive the hydrogel through a 27-gauge line. The texture analyzer was equipped with a fixed platform and a cylindrical acrylic probe with a diameter of 5 cm. The samples were transferred to a 1 mL syringe and the runs were made through a 27-gauge needle at a speed of 0.7 mm/s. Then, graphics were obtained with respect to forces as a function of distance.

#### 2.2.5. Dynamic Viscosity and Storage Module Measurement

Dynamic viscosity and storage modules were determined by using an Anton Paar MCR301 rheometer (Graz, Austria). The procedure involved a 5-cm cone plate measuring geometry and oscillatory shear responses (G′ or storage modulus and G″ or loss modulus), determined at 0.1 Pa over the frequency range of 0.1–100 rad s^−1^. Samples were evaluated in triplicate at 25 °C. The test conditions were within the linearity range of viscoelastic properties.

### 2.3. ISO Guinea Pig Maximization Sensitization Test

This test was performed according to ISO 10993-10 requirements, “Tests for irritation and skin sensitization.” The test hydrogel was extracted in 0.9% sodium chloride USP and sesame oil, NF. Each extract was intradermally injected and occlusively patched on ten test guinea pigs (per extract). The extraction vehicle was similarly injected and occlusively patched to five control guinea pig (per vehicle). Two induction phases were applied; induction II phase was applied 6 days after the completion of induction I. Following a recovery period (14 days after completion of Induction II), test and control animals received a challenge patch of the appropriate test article extract and vehicle control. All sites were scored for dermal reactions at 24 h and 48 h after patch removal. Dermal reactions were scored according to erythema in accordance with the criteria, and the grading scale is as follows: no visible change, 0; discrete or patchy erythema, 1; moderate and confluent erythema, 2; intense erythema and swelling, 3.

### 2.4. Rabbit’s Experimental Design

All procedures were carried out according to the Guide for the Care and Use of Laboratory Animals (NRC, 2011) and with the approval of the Institutional Ethics and Security Committee (Protocol Nº 237/15, 15 October 2015). The Center for Comparative Medicine (CMC) is in compliance with Good Laboratory Practices (GLP) for conducting preclinical tests and the certifications of local regulatory agencies.

**Figure 1:**
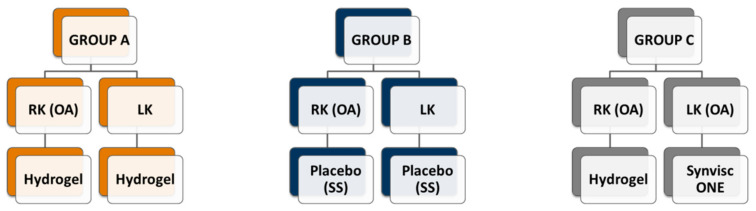
Test in vivo (rabbit model). RK: right knee; LK: left knee; OA: osteoarthritis generated by anterior cruciate ligament transection; SS: saline solution.

Forty-eight mature (4 months old) New Zealand white (NZW) rabbits, with a weight difference of less than 10%, were randomly divided into three experimental groups (see Figure 1), with 18 animals for each group A and B (9 males and 9 females, respectively) and 12 animals for a third group C (6 males and 6 females) [25,26]. Group A and B underwent unilateral anterior cruciate ligament transection (ACLT) of the right knee, while, in the left knee, a simulated surgery was performed as a control. Twenty-eight days after surgery, both knees of group A were treated with 0.3 mL of the injectable formulation under study. Meanwhile, both knees of group B were treated with 0.3 mL of sterile phosphate-buffered saline (placebo).

The animals of group C were surgically operated similarly; however, the induction of osteoarthritis was performed in both knees: 28 days after the intervention, 0.3 mL of HA was injected in the left knee and 0.3 mL of the injectable formulation under study was injected in the right knee.

The animals were kept under controlled environmental conditions: temperature at 22 ± 3 °C, controlled photoperiod (12 h of light, 12 h of darkness), and free access to commercial food and water.

#### 2.4.1. Surgical Procedure

An experimental model of osteoarthritis was developed according to that described by Yoshioka et al. [25] and Shimizu et al. [26]. All rabbits were anesthetized to perform a lateral approach to the right knee joint through a medial parapatellar incision. The tissue was subcutaneously incised along the same line to visualize the partition between the proximal surface of the fascia lata and the femoral bicep muscle and the distal surface of the lateral retinaculum. Finally, the fascia and joint capsule were incised to medially move the patella and to expose the cranial surface of the joint.

In this manner, the anterior cruciate ligament was visualized, which was transected with an Iris scissor. Subsequently, the articulation was instilled with sterile saline solution and sutured. The muscle, capsule, and synovium were sutured together using a suture polyglycolic acid 4-0 (Dexon Supralon, Buenos Aires, Argentina). The skin was sutured using continuous intradermal stitches with Nylon 30 or Nylon Surgical 3-0 (Ethilon, Ethicon, Raritan, NJ, USA).

In the left knee, the surgical intervention was simulated by performing the same procedure without cutting the cruciate ligaments.

A single administration was performed on each knee, 28 days after surgery. Thus, after trichotomy and local disinfection, the joint was placed in complete flexion, and the formulation was intra-articularly administered medially of the joint by using a 1 mL syringe with a 25/8 21Gx1 needle due to the characteristics of the test substance. As a precaution, the articular surface was not touched.

The animals were clinically evaluated each day, including an examination of the skin, mucous membranes, motor activity, and behavior patterns. In addition, they were studied for biochemical and hematological variables prior to surgical intervention 28 days after surgical intervention and at 3, 6, and 12 months after treatment.

#### 2.4.2. Radiological Studies

Radiological studies were carried out prior to the start of the study, 28 days after surgical intervention and 3, 6, and 12 months after treatment to determine the interarticular space (antero-posterior incidence). A range of ± 3 days was admitted in the execution of the studies. The plates were made by the personnel of Radiology Service of the Animal Health Hospital of the Faculty of Veterinary Sciences and digitized using the Image-Pro Plus 3.0.1 system (Media Cybernetics, Rockville, MD, USA) to determine the thickness (maximum, minimum, and average) of the inter-articular space for medial and lateral condyles in both knees.

#### 2.4.3. Euthanasia and Necropsy

Three animals of each sex of groups A and B were euthanized at 3, 6, and 12 months after treatment, while in group C, they were only euthanized at 6 and 12 months. Prior to complete necropsy, joint content was obtained and stored at −20 °C for further complementary analysis. Complete necropsy was carried out. Abdominal, thoracic, and cranial cavities were opened and internal organs were examined in situ. The necropsies included organ gross pathology, and blood and serum samples were taken for hematology and clinical chemistry. In addition, a histological study was conducted at the site of implantation and control, inguinal lymph node, liver, and spleen.

#### 2.4.4. Macroscopic Scoring

Prior to fixation in 10% buffered formalin, a macroscopic study was carried out by using the Indium ink technique [27]. By using the Image-Pro Plus system, the thickness of the joint capsule was determined.

Subsequently, the knees and joint capsules were analyzed according to the parameters indicated by Laverty et al. [27] with the following criteria: Grade 1 (intact surface): surface appears normal and does not retain any ink; Grade 2 (minimal fibrillation): site appears normal before staining, but retains Indium ink as elongated specks or light gray patches; Grade 3 (overt fibrillation): the cartilage is velvety in appearance and retains ink as intense black patches; Grade 4 (erosion): loss of cartilage exposing the underlying bone.

#### 2.4.5. Microscopic Scoring

The samples were fixed in 4% buffered formaldehyde for 8–10 h at 25 °C, washed in PBS, decalcified, dehydrated in an ascending series of ethanol, cleared in xylene, and finally embedded in paraffin. Sections (4 μm thick) were mounted on slides that were previously treated with 3-aminopropyltriethoxysilane (Sigma-Aldrich, St. Louis, MO, USA) and stained with hematoxylin-eosin. In the microscopic examination (blind to treatment) of the joint, the presence of inflammatory components, neovascularization, fibrosis, necrosis, and any other findings considered relevant was determined.

Subsequently, knees and joint capsules were analyzed according to the parameters showing the recommended grading system that captured the histological features of synoviopathy in OA [27].

Moreover, the femur was analyzed for degenerative changes in the articular cartilage; the findings were quantitatively assessed by using the scoring system described by Elmorsy et al. [28]. The femoral condyles were cut into four pieces (regions A, B, C, and D from lateral to medial condyle) along the sagittal plane, and all pieces were embedded in paraffin and stained with hematoxylin and eosin before quantitative assessment.

The right and left knees of each animal of the groups were analyzed and scored for each aspect; then, the score of each aspect was summed to obtain a total score for each knee.

#### 2.4.6. Hematology and Serum Chemistry

In order to test systemic effects, hematology and serum chemistry parameters were evaluated at baseline; at 1, 3, and 6 months; and before necropsy and blood samples were collected from animals by left ventricular puncture. Blood samples collected in EDTA tubes were analyzed by an automated hematology analyzer (Mindray BC-2800Vet, Shenzhen, China). The hematologic parameters, including red blood cells (RBCs), hematocrit (HCT), hemoglobin (HGB), mean cell volume of red blood cells (MCV), mean cell hemoglobin (MCH), mean corpuscular hemoglobin concentration (MCHC), white blood cells (WBCs) with differential count of heterophiles (HETs), eosinophils (EOS), basophils (BAS), lymphocyte (LYM), and monocyte (MNO), were analyzed. Blood was also taken for serum chemistry in glass tubes, then placed for approximately 30 min at ambient temperature, and serum was obtained by centrifugation at 3000× *g* rpm for 10 min. Clinical chemistry was determined with validated micro-methods for small volumes, with commercial kits (Wiener Labs, Rosario, Argentina) and a microplate reader (SPECTROStar Nano, BMG Labtech, Ortenberg, Germany). Blood urea nitrogen (BUN), creatinine (CRE), alanine aminotransferase (ALT), aspartate aminotransferase (AST), total protein (TP), albumin (ALB), and alkaline phosphatase (ALP) were measured.

### 2.5. Statistical Analysis

A comparative analysis was conducted between the different formulations, including statistically descriptive analysis, determination of normality, and analysis of variance (ANOVA). Later, a multiple-comparison test was performed to determine the effects of the treatments and associations between the studied variables. The different quantified parameters were evaluated by using the SPSS 10.1 programs (SPSS Inc., Chicago, IL, USA). The specific analyses of each variable are reported in Section 3. For all statistical analyses, *p* < 0.05 was considered significant. Results are expressed as mean ± standard deviation (SD).

## 3. Results and Discussion

### 3.1. Physicochemical Characterization of PQ10-CS-DVS Hydrogel

Each sample of PQ10-CS-DVS hydrogels was characterized in their spectroscopic, thermal, rheological, and texturometric properties. The last two assays were also evaluated before and after the heat-treatment of hydrogel to observe the potential differences produced by the sterilization process and to evaluate the generation of a compound with cytotoxic, genotoxic or mutagenic properties (Tables S2, S5 and S6, Supplementary Materials).

#### 3.1.1. FTIR Analysis

**Figure 2:**
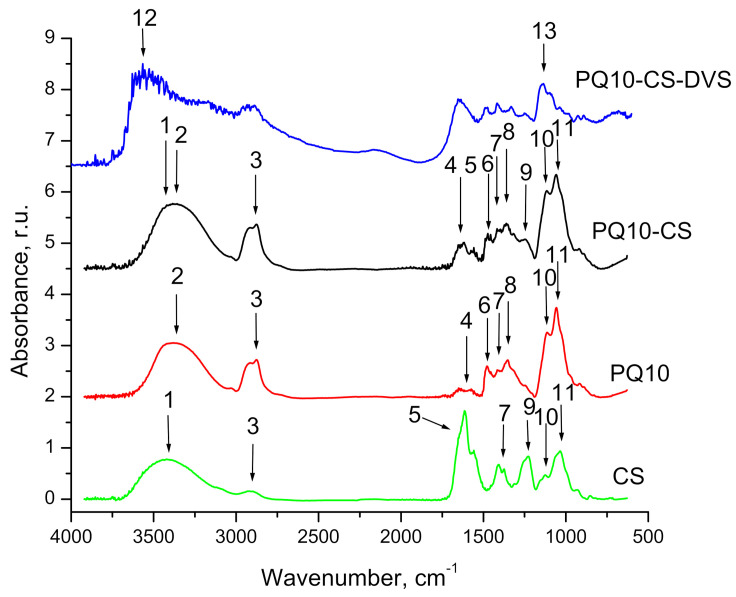
Infrared spectra of the crosslinked hydrogel compared with non-crosslinked gel and individual components taken as a reference. Numbers 1–11 means: **1:** 3423, str. O–H and, partially, N–H, (CS); **2:** 3383, str. O–H (PQ10); **3:** 2871, str. C–H (PQ10); 2906, str. C–H (CS); **4:** 1642 and 1577, quaternary ammonium group (PQ10); **5:** 1606, str. antis. –COO– (CS); 1556, Amide II (CS); **6:** 1477, –CH_3_ attached to the quaternary nitrogen (PQ10); **7:** 1411, str. sym. –COO– (CS); 1417, str. C–N (PQ10); **8:** 1352, def. O–H in the plane (primary and secondary alcohols) (PQ10); **9:** 1227, str. antis. SO_3_^−^ (CS); **10:** 1123, str. antis. C–O–S (CS); 1117, ether union (PQ10); **11:** 1057, str. C–O (primary and secondary alcohols) (PQ10); 1035, different vibrations of the pyranose ring (CS).

We used the spectroscopic method of FTIR to evaluate groups that appeared to be involved in the interaction of PQ10 and CS. Figure 2 shows the spectra of the crosslinked hydrogel (PQ10-CS-DVS, sample 3-3, see Table S1, Supplementary Materials), the non-crosslinked PQ10-CS complex, and the spectra of the pure substances, with PQ10 and CS as references. In the first case, the non-crosslinked PQ10-CS complex bands were assigned on the basis of the reference spectra of pure substances. Subsequently, they were compared with the spectrum of the crosslinked PQ10-CS-DVS complex, and differences were observed. From the analysis of the aforementioned spectra, the following bands were assigned for the non-crosslinked PQ10-CS complex:

Band assignment (cm^−1^): (str., Stretching; def., deformation; sym., symmetric; antis., anti-symmetric).

**1:** 3423, str. O–H and, partially, N–H, (CS); **2:** 3383, str. O–H (PQ10); **3:** 2871, str. C–H (PQ10); 2906, str. C–H (CS); **4:** 1642 and 1577, quaternary ammonium group (PQ10); **5:** 1606, str. antis. –COO– (CS); 1556, Amide II (CS); **6:** 1477, –CH_3_ attached to the quaternary nitrogen (PQ10); **7:** 1411, str. sym. –COO– (CS); 1417, str. C–N (PQ10); **8:** 1352, def. O–H in the plane (primary and secondary alcohols) (PQ10); **9:** 1227, str. antis. SO_3_^−^ (CS); **10:** 1123, *str. antis.* C–O–S (CS); 1117, ether union (PQ10); **11:** 1057, str. C–O (primary and secondary alcohols) (PQ10); 1035, different vibrations of the pyranose ring (CS).

It should be noted that the spectrum of the complex without crosslinking PQ10-CS has a similar pattern to that of the total bands of both pure components. The differences observed in the bands of the crosslinked complex in relation to the non-crosslinked ones are as follows.

**12:** 3383 cm^−1^, O–H vibration of polymeric association by hydrogen bridge in PQ10-CS, which decreases in PQ10-CS-DVS. Consequently, a band appears at 3562 cm^−1^ corresponding to O–H vibration joined by intramolecular hydrogen bridge; **13:** 1057 cm^−1^, CO vibration of the primary and secondary alcohol groups corresponding to PQ10, which decreases in the PQ10-CS-DVS, and a band appears at 1139 cm^−1^ (–COC–) that falls within the absorption zone of the ether groups as an indication of crosslinking.

From these results, we can conclude that the bands modified in the spectrum of the crosslinked complex (PQ10-CS-DVS) with respect to the complex without crosslinking (PQ10-CS) correspond to the functional groups involved in crosslinking, which are the –OH primary and secondary (bands of deformation), and the consequent formation of ether bonds –COC–. Bands corresponding to stretching –OH groups are also modified, since the level of packing acquired by the crosslinked complex allows for the formation of intramolecular hydrogen bridge junctions between the –OH groups, which remain unreacted.

#### 3.1.2. Differential Scanning Calorimetry (DSC)

**Figure 3:**
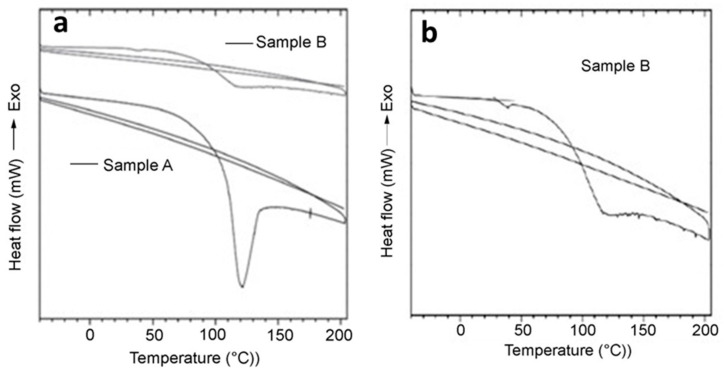
Differential scanning calorimetry (DSC) of hydrogels. (**a**) shows thermograms of sample 3-3 before (Sample A) and after (Sample B) lyophilization with three scans (heating/cooling/heating). (**b**) shows an expanded view of the thermal response (DSC) of sample B.

Figure 3a shows the thermograms corresponding to the hydrogel (PQ10-CS-DVS, sample 3-3, see Table S1, Supplementary Materials) before (Sample A) and after lyophilization (Sample B). Both samples present an endothermic event at around 100 °C due to the evaporation of water, but they are much more pronounced for the hydrogel of sample A. The loss of water in the lyophilized sample B indicates the presence of water that remains strongly associated, by hydrogen bonding, with macromolecular chains. Figure 3b shows an expanded view of the thermal response of the product of lyophilized sample B. The hydrogel of lyophilized sample B was studied by using this technique, since no glass transition temperature (T_g_) was observed in the hydrogel of sample A, possibly because the large amount of water masked the thermal events of macromolecules. However, in sample B, it was possible to observe a glass transition temperature (T_g_) at 29.9 °C. As shown in Figure 3a,b, the cooling and second heating stages for each sample showed no presence of transition.

#### 3.1.3. Thermogravimetric Analysis (TGA)

**Figure 4:**
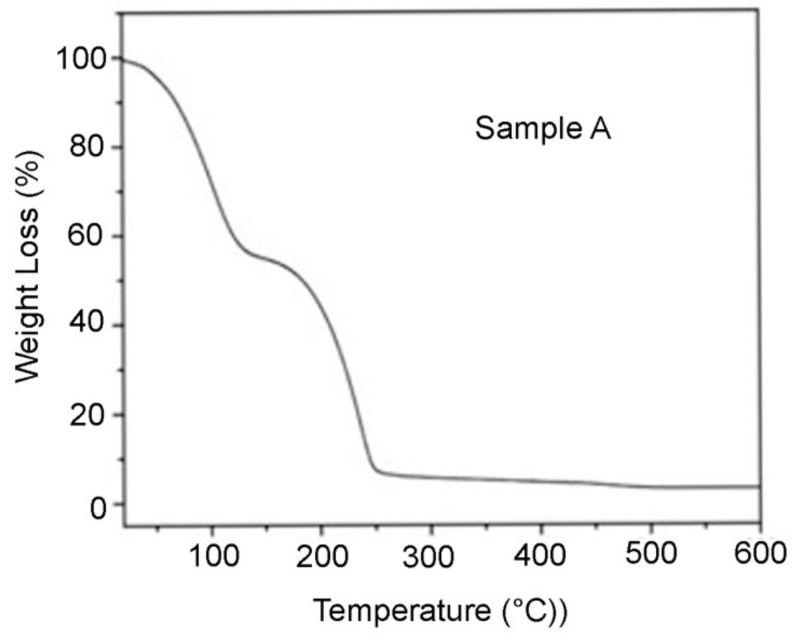
Thermogravimetric analysis (TGA) of hydrogel from sample 3-3 (Sample A) within a temperature range of about 23 °C to about 600 °C.

Figure 4 shows the thermogram generated in the analysis of the hydrogel PQ10-CS-DVS of sample A (Figure 3a, DSC). Onset temperature (T_o_) was determined by extrapolating the slopes before and after transition. The results show that below 150 °C, a 45% loss of mass was associated with the removal of water from the hydrogel material (T_o_ = 62.5 °C). From this temperature, up to 600 °C, a thermal event associated with the decomposition of the hydrogel material was observed. Sample A presents a T_o_ of 201.2 °C with a loss of mass of 49.4%. The total mass loss at 600 °C resulted in a residue of 3.0%.

#### 3.1.4. Rheology

In addition, as part of the evaluation, control samples were performed in order to observe changes produced in rheological properties when a covalent crosslinking reaction was carried out. Control samples consisted of the same combinations of polymers PQ10 and chondroitin 4-sulfate (CS) without the presence of the divinylsulfone crosslinking agent (i.e., PQ10 at 1–3% and PQ10 at 1–3% and CS at 0.1–0.3%, respectively).

The results obtained (Table S3, Supplementary Materials) show that the covalent crosslinking reaction of the cationic polymer with glycosaminoglycan provides a substantial increase in both dynamic viscosity and the storage module of the resulting hydrogel when compared to the control with a combination of the same polymers at the same concentrations without the covalent crosslinking reaction. In cases where the polymer PQ10 is more concentrated (from 2% *w*/*v*) and the initial concentration of the cross-linker is also higher (3–4% *w*/*w* with respect to the initial total mass of the polymers PQ10 and CS), we observed that hydrogel stiffening exceeds the measurement limits of the instrument. In this first rheological evaluation of hydrogels, we observed a wide variety of viscosities and modules, which depend on the initial concentration of PQ10 and the cross-linking agent.

#### 3.1.5. Thermal Stability of Covalently Crosslinked Hydrogel

Next, we evaluated the effect of thermal treatment on the stability of the rheological properties of the hydrogels (Table S4, Supplementary Materials) before and after sterilization cycle by autoclave at 121 °C for 15 min. From these results, we can conclude that the heating process reduces both viscosity and storage modulus and, consequently, extrudability force. This decrease seems to be more marked at a lower crosslinker percentage (i.e., 1 or 2%). However, when the percentage of crosslinking increases (i.e., 3 or 4%), the decrease observed in these parameters is now lower or almost is not modified.

Taken together, looking at both results, especially those of the extrusion force assays, we chose sample 3-3 (PQ10 3% *w*/*v*/CS 0.3% *w*/*v*/DVS 3% *w*/*w*) for in vitro biological tests, as it showed appropriate rheological properties and stability after the heating process (Figures S2 and S3, Supplementary Materials).

### 3.2. In Vivo Studies

The in vivo studies aimed to evaluate the safety and effectiveness of the cellulose-based hydrogel PQ10 (3%)–Chondroitin Sulfate (0.3%)–divinyl sulfone (3%) (PQ10-CS-DVS). These were performed using two experimental models, guinea pigs, and rabbits, and the results are described below. After analyzing different combinations of hydrogel concentrations, the one that showed the best stability profile after autoclaving was assessed by using rheological parameters. The selected sample did not show changes in elastic modulus, G′, after being subjected to the sterilization process (see Supplementary Materials, Table S4). On the other hand, TGA tests show that the degradation temperature of the hydrogel is 200 °C, well above the 120 °C of the autoclave.

#### 3.2.1. Sensitization Test

PQ10-CS-DVS was evaluated for its potential to cause delayed dermal contact sensitization in a guinea pig maximization test. The results showed that there was no evidence of delayed dermal contact sensitization (score = 0 for all extract tested). Thus, the formulation under study was not considered a sensitizer in the guinea pig maximization test.

#### 3.2.2. Implantation of PQ10-CS-DVS in Rabbits’ Knees

PQ10-CS-DVS was implanted in the knee of rabbits to evaluate the safety of the substance for the treatment of osteoarthritis. Subjects were divided into three groups (see Figure 1): Group A underwent unilateral ACLT of the right knee, while, in the left knee, a sham surgery was performed as a control; both knees were treated with 0.3 mL of the injectable formulation under study. Group B also underwent unilateral ACLT of the right knee and a sham surgery in the left knee, but both knees were treated with 0.3 mL of sterile phosphate-buffered saline (placebo). However, group C had ACLT surgery in both knees and was treated with 0.3 mL of our formulation in the right knee and 0.3 mL of commercial HA (Synvisc One) in the left knee.

After implantation, all specimens were evaluated by conducting radiological, macroscopic, and histopathological studies at 3, 6, and 12 months.

##### Radiological Studies

In order to evaluate the effect of both implantable hydrogels in the knees, a digital morphometric study was performed on radiographic plates to determine the following measurements of the inter-articular space: minimum and maximum separation between the lateral condyles of the right and left knees; minimum and maximum separation between the medial condyle of the right and left knees. Then, a statistical analysis of all these variables was carried out. Table 1 shows the average values of the separation between the medial condyle of the right and left knees (R-MC and L-MC, respectively) and of the separation between the lateral condyles of the right and left knees (R-LC and L-LC, respectively) from Groups A, B, and C.

In a comparative analysis of the results obtained between both knees of the three groups, it can be concluded that no differences were found to be attributable to the treatments. In Figure S4 (Supplementary Materials), representative plates for the three groups at different times are shown.

##### Macroscopic Evaluation

**Figure 5:**
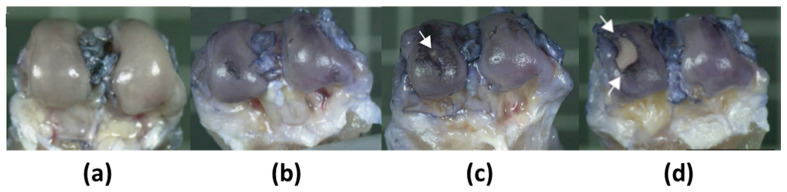
Different scores of Indium ink test. (**a**) Grade 1: cartilage intact surface, normal surface, and no retention of Indium ink. (**b**) Grade 2: cartilage minimal fibrillation; surface retains Indium ink as elongated specks or light-gray patches. (**c**) Grade 3: cartilage overt fibrillation; areas velvety in appearance and retains Indium ink as intense black patches. (**d**) Grade 4: cartilage erosion; loss of cartilage exposing the underlying bone.

Complete postmortem examination, including a gross assessment of all major organs, revealed no tissue change associated with treatments. The findings were evaluated according to classification grades 1 to 4, as explained above [27]. Figure 5 shows how the Indium ink is retained when cartilage damage is present, and four cases are illustrated, with different levels of cartilage damage.

Table 2 shows the average results, discriminated by group and time of analysis; that is, the analysis of paired samples detected differences between the right and left knees in the animals of groups A and B (3, 6, and 12 months). The right knee showed a higher score (cartilage damage) than compared to the left knee. These findings are in accordance with experimental design, where only the right knee had induced osteoarthritis.

Another analysis was performed for independent samples, and no differences were found between the same knees of different groups.

At 6 months, no significant differences were detected between the right and left knees of the different groups. However, at 12 months, significant differences were observed between the right and left knees of both groups A and B. It is notable that the test substance did not significantly increase the damage to the cartilage caused by the experimental model when compared with the placebo group until 6 months, and the differences evidenced at 12 months could be attributed to the length of the trial and final age of the animals.

In the case of group C, after an analysis of paired samples, it was observed that no differences were observed between the right and left knees at 6 months. At 12 months, when comparing the right and left knees (*p* = 0.055), more cartilage damage was observed for the left knee (knee injected with HA).

##### Histopathological Study

Table 3 shows the results obtained from sampling in joint capsules and microscopic scoring according to Elmorsy et al. [28]. In this case, an analysis of paired samples was carried out, with no significant differences detected in the analyzed variables between the right and left knees.

An analysis of independent samples was also performed; the variables were analyzed for each knee (right and left), for each group (A, B), and at different sampling points (3, 6, and 12 months). At 3 months, there was a tendency towards a greater proliferation of blood vessels of the right knee in group B (placebo group) in relation to group A (experimental group). Moreover, at 12 months, an increase in fibroblast/fibrocyte proliferation was observed in the left knee in relation to group A (Table 3).

Group C was analyzed as paired samples since both knees had induced OA, but the right knee was injected with hydrogel PQ10-CS-DVS, and the left knee was injected with commercial HA. At 6 months and 12 months, no differences were observed between the right and left knees. The hydrogel under study did not produce additional changes in the synovial membrane with respect to commercial HA.

Table 4 shows histopathological results for the rabbit femurs of groups A, B, and C at different sampling times (3, 6, and 12 months). Degenerative changes in the articular cartilage were quantitatively evaluated according to the scores provided by Laverty et al. [27]. The sum of all the aspects was measured, and the average for the right and left knees of the animal of each group was analyzed.

From the results shown in Table 4, it can be observed that, at 3 months, no significant differences were observed in groups A and B. On the other hand, some differences were observed in group A between the right and left knees at 6 and 12 months, although they were not observed when comparing the same knee from different groups. The greater value obtained for the right knee when compared to left knee indicated greater damage to the cartilage; this is in agreement with the model adopted in the study, where only the right knee had OA induced by an ALCT.

Student’s *t*-test was performed for independent samples, with a significant difference observed in the right knees between groups A and B, which is possibly attributable to the treatment.

Based on the information collected in this study, it can be concluded that the formulation PQ10-CS-DVS is harmless until 12 months of sampling, since no alterations in the evaluated biochemical and hematological parameters were found to be associated with treatment, demonstrating short-term and long-term systemic safety in the dose used.

In the case of group C, where both knees had induced OA, the histological results of the femur showed a significant difference between the left and right knees at 12 months. For the left knee, significant cartilage damage was observed at 12 months when compared to the right knee. This could be attributed to the stability of the injected compounds. HA is well known to last up to 10–12 months before biodegrading. According to the results shown in Table 4, at 6 months, both knees showed similar cartilage damage, probably due to the presence of the viscosupplements in the synovial liquid, whereas, at 12 months, significant cartilage damage was observed only for the left knee, probably due to the disappearance of the HA viscosupplement. However, the right knee injected with hydrogel PQ10-CS-DVS did not show any significant differences between 6 and 12 months. In order to demonstrate that these findings were due to the presence of the hydrogel PQ10-CS-DVS at 12 months, synovial liquid was extracted and stained with hematoxylin-eosin to detect the presence of the hydrogel. As a positive control, the synovial liquid of the animal implanted with PQ10-CS-DVS was analyzed 24 h after implantation and, as a negative control, the synovial liquid of an animal injected with saline solution was also analyzed 24 h after injection.

**Figure 6:**
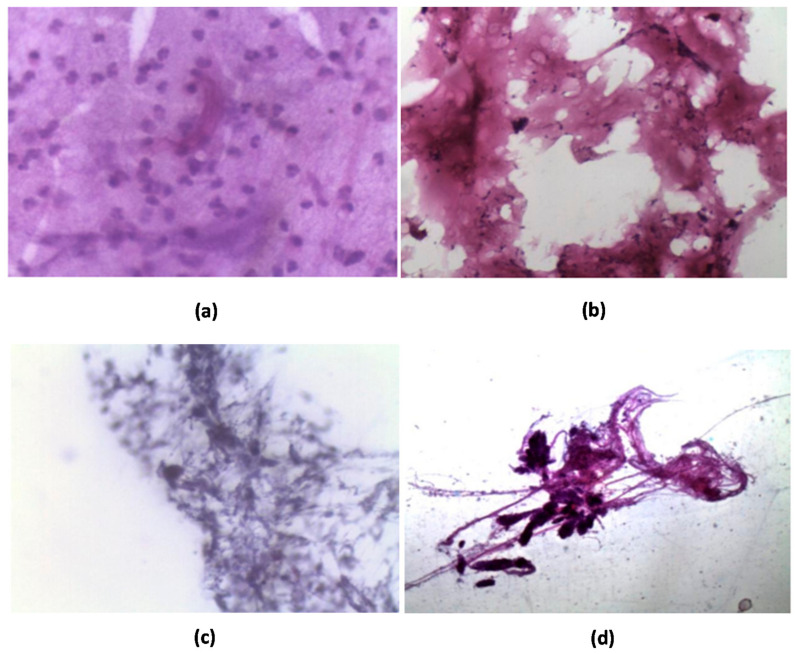
Images (**a**,**b**) correspond to positive control. (**a**) Large amount of basophilic material, slightly translucent, with a large cellular component, composed mainly of polymorphonuclear leukocytes, and predominantly heterophiles. In isolation, traces of an eosinophilic, amorphous, fibrillar, and acellular material can be found. (**b**) A fibrillar material, amorphous, eosinophilic, acellular, and in large quantities. (**c**) Negative control shows material that is slightly basophil, which has very fine fibers, forming networks, with few cells between their fibers. The material can be compatible with synovial fluid of the joint. (**d**) Synovial fluid extracted at a sampling point of 12 months shows fibrillar material, eosinophil, cell inflammation, and mostly composed of heterophilic polymorphonuclear cells and lymphocytes.

Figure 6 shows histopathological images for a 12-month sampling period of synovial liquid and negative and positive controls. As shown in the images, the hydrogel was observed in synovial liquid extracted at a sampling point of 12 months (Figure 6d and positive control Figure 6a,b). According to these findings, the greater stability of the hydrogel PQ10-CS-DVS compared to commercial HA favors the preservation of the cartilage. It is important to highlight that the selection of PQ10 as a biopolymer for this application was due to several factors: 1. Biocompatibility was demonstrated in a study in which PQ10 injected into a rabbit eye showed no inflammatory responses or altered retinal activity as the HA gold standard [16]. 2. The PQ10 biopolymer is a polycationic molecule that contains a quaternary amino group, where the charge of these positive groups remains unchanged regardless of the changes that may occur in the pH of the environment due to changes in the biological activity of the cells. 3. The polycationic surface of the polymer is capable of strongly interacting with the well-known electronegative charge present on the surface of cells through electrostatic interaction. 4. PQ10 is a polymer of plant origin that is not easily degraded by enzymes of animal origin, since humans do not have cellulase enzymes. 5. The high thermal stability of PQ10 increases when the polymer is covalently crosslinked. It is well known that HA injected into a knee with OA is degraded by an endogenous natural enzyme hyaluronidase released by inflammatory cells present in the affected articulation, as well as by reactive oxygen species (ROS) generated in the inflammatory site, limiting its period of intra-articular residence [29]. In a study in rabbit knees, it was shown that the apparent half-life of Hylan G-F 20 in the joint was 8.8 days, and more than 95% of the total Hylan was cleared from the joint by 4 weeks [30]. Another study shows that Durolane™(HA) has a half-life of 32 days in the rabbit knee joint [31]. In recent years, a so-called “one-shot” HA has been developed. Most “one-shot” preparations contain crosslinked HA, such as Synvisc One (Hylan G-F20), which is known to have longer residence times within the joint compared to HA that is not chemically modified [32]. It being crosslinked makes it a good candidate for comparison with our hydrogel PQ10-CS-DVS.

In this study, the degradation of PQ10-CS-DVS was not analyzed, but it was demonstrated that, at 12 months, the substance was present in the synovial fluid of the knees of the rabbits. The presence in the joint of the hydrogel PQ10-CS-DVS at 12 months could explain its greater chondroprotective effect compared to Hylan G-F 20 (Synvisc One).

Based on the information collected for group C until 12 months, there was evidence of greater cartilage damage and femur in the left knee treated with HA (Synvisc one) [9] compared to the right knee treated with PQ10-CS-DVS at the final sampling point of 12 months. These long-term effects are related to the biostability of the substance, as explained above. Hydrogel PQ10-CS-DVS in the synovial liquid of the treated animal at 12 months could protect the cartilage from progression of OA.

**Figure 7:**
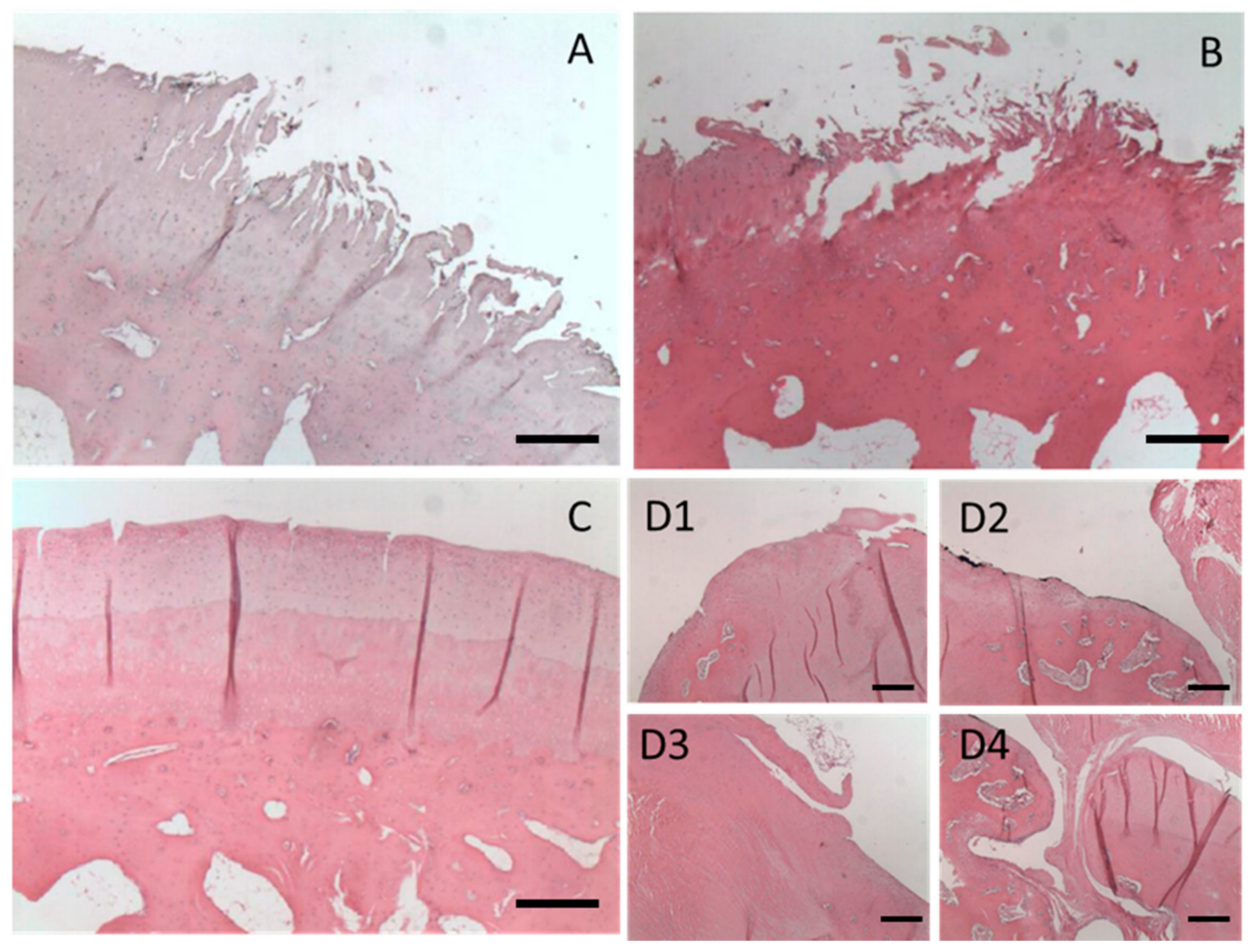
Representative histopathology of femur, Group C, 40×. (**A**) Right femur, 6 months: a focal region of marked fibrillations of the articular cartilage is observed. (**B**) Left femur, 6 months: an extensive focal region of articular cartilage erosion is observed, exposing the subchondral bone and numerous fibrillations and fissures at the edges of the said region. (**C**) Right femur, 12 months: numerous fissures are observed in the hyaline cartilage, which is clearly eroded in its articular surface. The underlying bone presents mild thickening in its architecture (bone remodeling). (**D1**–**D4**) Four independent images of the same left femur, 12 months: exuberant lesion, with extensive focal loss of articular cartilage. The components of an old repair of a bone fracture are observed. Greater proliferation of fibrocartilage, bone, and newly formed vessels towards the depth of the femur. Complete loss of the architecture of the articular surface, with marked perivascular fibrosis, covering the joint face of the formed callus. Scale bar: 500 μm.

Figure 7 shows an example of group C, where the right and left femurs are compared at 6 months and 12 months. In these results, increased damage can be observed on the left femur (Synvisc ONE) in comparison with right femur (Hydrogel PQ10-CS-DVS).

In addition, from the histopathological study of the implantation site, the inguinal lymph nodes, liver, and spleen were studied. General histopathology showed lesions in different organs, most of which were chronic, background lesions in animals of the same age as those used, considering the length of the trial (12 months) and the age of the animals from the CMC colony. Moreover, there were no signs of migration of the test substances.

##### Hematology and Serum Chemistry

The treated groups showed no significant (*p* > 0.05) changes in hematological parameters when we compared the values in each sample at baseline within the 12 months of observation (Dunnett test; see Table S7, Supplementary Materials). The biomarkers, such as RBCs, HCT, HGB, MCV, MCH, MCHC, PLT, WBCs, HETs, EOS, BAS, LYM, and MNO counts in treated groups, were in the normal range compared to the control group. Therefore, no treatment-related changes were observed in the biochemical profile (BUN, CRE, ALT, AST, TP, ALB, and ALP) (*p* > 0.05; Table S8, Supplementary Materials).

Based on the information collected in this study, it can be concluded that the formulation PQ10-CS-DVS is harmless until 12 months of sampling, since no alterations in the evaluated biochemical and hematological parameters were detected to be associated with the treatment, demonstrating short-term and long-term systemic safety in the used dose.

## 4. Conclusions

This study demonstrated biological safety at short-term and long-term use of hydrogel PQ10-CS-DVS. In vivo studies have demonstrated its safety in terms of sensitization, systemic toxicity, and migration.

An animal model with induced OA was studied to assess local effects in knee cartilage and synovial capsule using saline solution (placebo) and HA “Synvisc One” as a reference. The durability of the new hydrogel and its local effects were superior to HA in the long term. From this comparative study, it was concluded that the hydrogel (PQ10-CS-DVS) in the synovial liquid at 12 months protects the cartilage from OA progression.

## Figures and Tables

**Table 1:** Descriptive statistics of the radiological study for male rabbits.

Group	R-LC	R-MC	L-LC	L-MC	Time
A	1.586 ± 0.615	1.131 ± 0.223	1.564 ± 0.680	1.122 ± 0.171	0
B	1.199 ± 0.478	1.039 ± 0.139	1.232 ± 0.402	1.034 ± 0.328	0
A	0.744 ± 0.340	1.221 ± 0.157	0.859 ± 0.324	1.369 ± 0.319	28 d ^1^
B	1.171 ± 0.908	1.082 ± 0.382	0.866 ± 0.797	0.992 ± 0.206	28 d
A	1.601 ± 0.291	1.226 ± 0.366	1.312 ± 0.348	1.115 ± 0.394	3 m ^1^
B	1.569 ± 0.921	1.080 ± 0.375	1.486 ± 1.022	0.996 ± 0.451	3 m
A	1.510 ± 0.440	1.149 ± 0.430	1.724 ± 0.517	0.838 ± 0.400	6 m
B	1.220 ± 0.477	1.001 ± 0.503	1.313 ± 0.490	0.673 ± 0.181	6 m
A	0.909 ± 0.354	0.589 ± 0.155	2.662 ± 3.510	0.812 ± 0.459	12 m
B	0.971 ± 0.094	1.006 ± 0.179	1.080 ± 0.270	0.656 ± 0.473	12 m
C	1.362 ± 0.438	1.150 ± 0.239	1.302 ± 0.472	1.155 ± 0.271	0
C	0.982 ± 0.265	1.143 ± 0.230	1.178 ± 0.531	0.953 ± 0.169	28 d
C	1.782 ± 0.390	0.866 ± 0.611	1.634 ± 0.587	1.115 ± 0.573	3 m
C	1.074 ± 0.395	0.843 ± 0.354	1.223 ± 0.412	1.050 ± 0.108	6 m
C	0.629 ± 0.161	0.601 ± 0.209	0.782 ± 0.262	0.869 ± 0.366	12 m

^1^ d = days; m = months.

**Table 2:** Macroscopic evaluation; Indium ink method.

Group	Average Score ^1^		Time (Months)
	Right Knee	Left Knee	
A	2.00 ± 1.22	2.40 ± 1.14	3
B	2.66 ± 0.81	2.00 ± 1.41	3
A	3.16 ± 0.98	2.17 ± 1.47	6
B	3.20 ± 1.64	2.16 ± 1.17	6
A	3.83 ± 0.40	2.00 ± 1.09	12
B	3.00 ± 1.09	1.33 ± 0.51	12
C	2.80 ± 1.30	3.00 ± 1.41	6
C	2.33 ± 1.50 ^#^	3.33 ± 0.81 ^#^	12

^1^ N = 6. ^#^ *p* = 0.055. Group A: PQ10-CS-DVS hydrogel is implanted in both knees (right knee: induced osteoarthritis (OA); left knee: sham control). Group B: phosphate-buffered saline is injected in both knees (right knee: induced OA; left knee: sham control). Group C: right knee: PQ10-CS-DVS hydrogel, left knee: hyaluronic acid Synvisc one hydrogel. Both knees with induced OA. PQ10: Polyquaternium-10; CS: chondroitin sulfate; DVS: divinylsulfone.

**Table 3:** Histopathological assessment of osteoarthritis synoviopathy for different experimental groups.

Criteria	12 Months	Group A		Group B		Group C	
		R	L	R	L	R	L
Synoviocytes	Proliferation	0.50 ± 0.55 ^1^	0.33 ± 0.52 ^1^	0 ^1^	0.17 ± 0.41 ^1^	1.00 ± 0.63	1.17 ± 0.75
	Hypertrophy	0.50 ± 0.55	0.67 ± 0.52	0.67 ± 0.52	0.67 ± 0.52	0.67 ± 0.52	0.83 ± 0.41
Inflammatory infiltrate	Granulocytic infiltrate	0	0	0	0	0	0
	Fibrinous exudate	0	0	0	0	0	0
	Lymphoplasmacytic infiltrate	0	0	0	0	0	0
	Lymphoplasmacytic aggregates/follicles	0	0	0	0.17 ± 0.41	0	0
Synovial stroma	Villous hyperplasia	1.17 ± 0.98	1.50 ± 0.55	1.17 ± 0.98	0.83 ± 0.75	2.33 ± 0.52	2.00 ± 1.10
	Proliferation of fibroblasts/fibrocytes	2.33 ± 0.82	1.83 ± 0.41^1^	2.00 ± 0.89	2.67 ± 0.52^2^	2.00 ± 0.63	2.50 ± 0.55
	Proliferation of blood vessels	1.00 ± 0.63	0.83 ± 0.75	0.50 ± 0.55	0.83 ± 0.41	0.83 ± 0.41	0.83 ± 0.41
	Cartilage/bone-detritus	0	0.17 ± 0.41	0.50 ± 1.22	0.33 ± 0.82	0	0.50 ± 1.22
	Hemosiderosis	0	0	0	0	0	0

^1^ Different numbers indicate significant differences *p* < 0.05 within the values of the left knee. R: right knee; L: left knee. Scoring: 0, normal; 1, slight; 2, moderate; 3, severe.

**Table 4:** Histopathological results of femur.

Group	Time (Months)	Average Score	
		Right Knee	Left Knee
A	3	55.17 ± 21.08	50.00 ± 10.41
B	3	65.00 ± 16.19	54.67 ± 8.33
A	6	76.50 ± 25.70	58.17 ± 17.55
B	6	78.83 ± 16.92	65.80 ± 15.00
A	12	96.00 ± 20.13	48.83 ± 15.38
B	12	65.50 ± 19.37	53.67 ± 22.18
C	6	77.17 ± 17.51	86.00 ± 14.64
C	12	68.00 ± 31.20	92.67 ± 23.83 ^1^

^1^ *p* < 0.01.

## Data Availability

The data presented in this study are available in the article.

## Supplementary Materials

The following are available online at https://www.mdpi.com/article/10.3390/polym13244426/s1. Table S1: Hydrogels of PQ10-CS with DVS at different concentrations, Table S2: Test system mammalian cell culture scoring, Table S3: Dynamic viscosity and elastic module of PQ10-CS-DVS hydrogels, Table S4: Analysis of stability of covalently crosslinked hydrogel reaction products before and after heating process, Table S5: Test article to negative control comparison, Table S6: Positive to negative control comparison, Table S7: Hematology parameters, Table S8: Serum chemistry data, Figure S1: Chemical structure of chondroitin sulfate (CS) as chondroitin 4-sulfate (C-4S), Polyquaternium-10 (PQ10) and divinylsulfone (DVS), Figure S2: Typical rheological profiles of G′ (storage modulus), G″ (loss modulus), and η* (complex viscosity) for hydrogel PQ10 (3% *w*/*v*)/CS (0.3% *w*/*v*)/DVS (3% *w*/*w*) (sample 3-3, Table S4) after heating process, Figure S3: Viscosity profile (η) vs. Share Rate (γ) for hydrogel PQ10 (3% *w*/*v*)/CS (0.3% *w*/*v*)/DVS (3% *w*/*w*) (sample 3-3, Table S4) after heating process, Figure S4: Representative radiological plates in rabbits from groups A, B, and C at different times 0, 6, and 12 months, respectively.
