# Supplementary material for: Biocompatible Hydrogel for Intra-Articular Implantation Comprising Cationic and Anionic Polymers of Natural Origin: In Vivo Evaluation in a Rabbit Model
Source: Polymers (Basel). 2021 Dec 16;13(24):4426. doi: 10.3390/polym13244426 (PMC8707494; doi:10.3390/polym13244426)

Chemical characterization of extractables from PQ10-CS-DVS hydrogel

PQ10-CS-DVS hydrogel contained in a polycarbonate syringe was extracted in purified water, dichloromethane and hexane. The Non-Volatile Residue (NVR) was determined. The resulting extracts were analyzed by Fourier Transform Infrared Spectroscopy (FTIR), Gas Chromatography-Mass Spectrometry (GC-MS) and Ultra Performance Liquid Chromatography-Mass Spectrometry (UPLC-MS). The purified water test extract was additionally analyzed by Inductively Coupled Plasma-Mass Spectrometry (ICP-MS) and GC-MS Headspace. These studies were developed in the certified laboratory NAMSA (USA) under the standard ISO 10993-18-Chemical Characterization.

The purpose of this study was to perform a chemical characterization of extractable and leachable compounds from extracts of PQ10-CS-DVS hydrogel in: purified water, dichloromethane and hexane, according to ISO 10993-18. In this case, we used it as part of an assessment of the overall biological safety of hydrogel as a potential medical device (ISO 10993-1 and ISO 14971). We also measured the level of leachable substance in the hydrogel so as to allow the assessment of compliance with the allowable limit derived in that substance from health-based risk assessment (ISO 10993-17). First, an exhaustive extraction was performed according to ISO-10993-12. In the case of purified water, 5 extraction cycles were needed to obtain the endpoint with 87.1 mg of Non-Volatile Residue (NVR). However, only 2 cycles were required for dichloromethane and hexane extracts with 0.3 and 0.1 mg of NVR, respectively.

In the analysis by FTIR, no bands were detected in any extracts. However, analyzed by GC-MS, the extracts yielded zero semi-volatile compounds greater than the quantitation limit for the hexane test article. For dichloromethane extract, however, 1,4-oxathiane, 4,4-dioxide (28 μg/test article; 13.44 minutes retention time) appeared. This compound is probably formed by degradation of divinylsulfone during the heating process. Other compounds also appeared at the trace level (~ μg), such as cyclotetra, cyclopenta and cyclohexa-siloxanedodecamethyl in the purified water extract, probably leaching from the syringe material.

In the ICP-MS technique, only Mg, Al and Zn metals coming from commercial chondroitin appeared at the trace level (~ μg) from a list of 47 metals analyzed. The presence of these metals in the final hydrogel comes from the raw materials. The ICP-MS of raw materials showed that PQ-10 had 6.2 ppm of Mg, 1.3 ppm of Al and <0.4ppm of Zn, whereas CS material had 49.3 ppm of Mg, 10.6 ppm of Al and 4.6 ppm of Zn.

In the case of UPLC-MS, there were no non-volatile compounds greater than the quantitation limit.

Finally, GC-MS Headspace technique was performed in order to detect solvent residues. The results showed absence of volatile compound residues of a list of 40 hazardous solvents analyzed; only a methyl silyl derivative (4.78 μg / test article) was detected in the purified water extract.

In-vitro cytotoxicity

Test System and Justification of Test System Mammalian cell culture monolayer consisting of L-929 mouse fibroblast cells (ECACC Cat #85103115, or equivalent source) was used. L-929 mouse fibroblast cells were propagated and maintained in flasks containing MEM at 37 °C with 5% carbon dioxide (CO_2). For this study, cells were seeded in 10 cm^2 cell culture wells, labeled with passage number and date, and incubated at 37 °C in the presence of 5% CO_2 to obtain sub confluent monolayers of cells prior to use.

A single preparation of the hydrogel was extracted in Minimum Essential Medium (MEM) at 37 °C for 24 h. The negative control, reagent control and positive control were similarly extracted. For test procedure, we used triplicate monolayers of L-929 mouse fibroblast contained sub confluent cell monolayers. The growth medium was replaced with 2.0 mL of the test extract in each well. Similarly, the growth medium in triplicate 10 cm^2 wells was replaced with 2.0 mL of the reagent control, the negative control and the positive control extracts. The wells of each plate were labeled with the appropriate lab number or control and the replicate number. Each plate was labeled with the test code and dosing date. The wells were incubated at 37 °C in 5% CO_2 for 48 h. Following incubation, the cells were examined microscopically (100X) to evaluate cellular characteristics and percent lysis. The evaluation was performed according to the test scoring presented in Supplementary Table S2.

The purpose of this study was to determine the potential of PQ10-CS-DVS hydrogel extract to cause cytotoxic effects using an in vitro mammalian cell culture test. This study was conducted following the guidelines of ISO 10993-5: Biological evaluation of medical devices. Part 5: Tests for in vitro cytotoxicity. A single preparation of the hydrogel was extracted in Minimum Essential Medium (MEM) at 37 °C for 24 h. The negative control, reagent control and positive control were extracted in the same way. Triplicate monolayers of L-929 mouse fibroblast cells were doused with each extract and incubated at 37 °C in the presence of 5% CO_2 for 48 h. Following incubation, monolayers were examined microscopically for abnormal cell morphology and cellular degeneration. The hydrogel extract met the requirements of the test since citotoxicity observed was less than grade 2 (mild reactivity, see Table S2), showing no evidence of causing cell lysis or toxicity.

In-vitro bacterial reverse mutation study

This test was performed according to ISO 10993-3, Tests for genotoxicity, carcinogenicity and reproductive toxicity, and OECD 471, Guideline for Testing of Chemicals, Bacterial Reverse Mutation Test.

Tubes containing molten top agar were inoculated with culture from one of the five tester strains, along with DMSO or saline extract. An aliquot of sterile water for injection or rat liver S9 homogenate was added, providing metabolic activation. The mixture was poured across triplicate plates. Parallel testing was conducted with negative controls (extraction vehicle alone) and positive controls (sodium azide, methyl methanesulfonate, 2-aminoanthracene, benzo pyrene, 2-nitrofluorene and ICR-191). The mean number of revertants for test extract plates was compared to the mean number of revertants of negative control plates for each of the five tester strains.

For DMSO and saline test extracts to be evaluated as a test failure or "potential mutagen", there must have been a 2-fold or greater increase in the number of mean revertants over the means obtained from the negative control strains TA98, TA100 and WP2*uvr*A and /or a 3-fold or greater increase in the number of mean revertants over the means obtained from the negative control for strains TA1535 and TA1537. Calculation of fold increase is the mean number of revertants of the test divided by the mean number of revertants for the respective negative control. Each positive control mean must have exhibited at least a 3-fold increase over the negative control mean, irrespective of vehicle, for all five tester strains. The negative control results of each tester strain should exhibit a characteristic number of spontaneous revertants.

Finally, the purpose of this study was to determine whether the hydrogel extract had the potential capability of causing genotoxicity. The bacterial reverse mutation study evaluated the potential of introducing mutagenic changes in specific selected bacterial strains. The test article PQ10-CS-DVS was evaluated for the potential of producing mutagenic changes at the histidine locus of the *Salmonella typhimurium* TA98, TA100, TA1535 and TA1537 tester strains or at the tryptophan locus of the *Escherichia coli* WP2*uvrA* tester strain, in the presence and absence of metabollic activation S9. For DMSO and Saline test extracts, in no case there was a 2-fold or greater increase in the mean number of revertants for TA98, TA100 and WP2*uvr*A tester strains or a 3-fold or greater increase in the number of mean revertants for TA1535 and TA1537 tester strains, as shown in Table S5. The negative control results for each tester strain exhibited a characteristic number of spontaneous revertants. Each positive control mean exhibited at least a 3-fold increase over the respective negative control mean for each of the five tester strains (see Table S6).

The main conclusion of this study is that DMSO and saline test extracts were considered non-mutagenic to *S. typhimurium* TA98, TA100, TA1535 and TA1537 tester strains and to *E. coli* WP2*uvrA* tester strain.

Supplementary Table S1. Hydrogels of PQ10-CS with DVS at different concentrations.

Sample	PQ10	CS	DVS
	%w/v	%w/v	%w/w[a]
1-1	1	0.1	1
1-2	1	0.1	2
1-3	1	0.1	3
2-1	2	0.2	1
2-2	2	0.2	2
2-3	2	0.2	3
3-1	3	0.3	1
3-2	3	0.3	2
3-3	3	0.3	3
4-3	4	0.4	3
4-4	4	0.4	4

[a]Weight percentage of crosslinking agent with respect to polymer total mass.

Supplementary Table S2. Test system mammalian cell culture scoring.[a]

Grade	Reactivity	Conditions of all Cultures
0	None	Discrete intra cytoplasmic granules, no cell lysis, no reduction of cell growth were found.
1	Slight	No more than 20% of the cells were round, loosely attached and without intra cytoplasmic granules, or showed changes in morphology; occasional lysed cells were present; only slight growth inhibition was observed.
2	Mild	No more than 50% of the cells was round, devoid of intra cytoplasmic granules; no extensive cell lysis; no more than 50% growth inhibition was found.
3	Moderate	No more than 70% of the cell layers contained rounded cells or was lysed; cell layers were not completely destroyed, but more than 50% growth inhibition was observed.
4	Severe	Nearly complete or complete destruction of the cell layers was found.

[a]This study was performed following ISO 10993-5-Cytotoxicity Testing.

Supplementary Table S3. Dynamic viscosity and elastic module of PQ10-CS-DVS hydrogels.

Sample	Dynamic viscosity (Pa) at different shear rates (s^{-1})				Storage Module (G', Pa) at different frequencies (Hz)		
	**0.01**	**0.46**	**10**	**100**	**0.1**	**4**	**40**
PQ10 1%[a]	18.6	8.9	2.2	0.54	0.52	9.8	33.4
PQ10 1%/CS 0.1%[a]	133	17.0	2.6	0.59	4.7	19.1	41.2
1-1[c]	441	54.0	4.8	0.72	6.8	27.2	44.3
1-2[c]	661	67.0	5.6	0.61	14.5	44.4	63.6
1-3[c]	3436	84.0	6.4	0.68	14.1	34.2	49.9
PQ10 2%[a]	89	30.0	6.0	1.2	4.1	37.4	106
PQ10-2%/CS 0.2%[a]	665	76.0	9.7	1.7	31.8	107	192
2-1[c]	2334	236	12.0	0.62	76.8	279	387
2-2[c]	75586	95.7	12.4	0.51	104	532	789
2-3[b-c]	-	-	-	-	-	-	-
PQ10 3%[a]	509	105	16.6	2.8	21.9	122	278
PQ10 3%/CS 0.3%[a]	1793	157	18.1	2.9	68.1	239	419
3-1[c]	67640	590	23.0	0.80	119	419	655
3-2[c]	40389	711	8.0	0.48	158	343	467
3-3[b-c]	-	-	-	-	-	-	-
4-3[c]	54922	2214	84.0	2.3	-	790	813
4-4[b-c]	-	-	-	-	-	-	-

[a]Control sample without crosslinking. Percentage given in (w/v) units.

[b]Highly rigid hydrogel. Viscosity and module exceeded the measurement limit of the instrument.

[c]Samples of PQ10-CS-DVS from Table S1.

Supplementary Table S4. Analysis of stability of covalently crosslinked hydrogel reaction products before and after heating process.[a]

Sample	Dynamic viscosity at 0.01 s^{-1} (Pa)		Storage Module at 40 Hz (Pa)		Extrusion force (g)[b]	
	Before	After	Before	After	Before	After
1-1	441	1	44	0.10	500	150
1-2	661	91	64	0.32	900	250
1-3	3436	173	50	1.80	1600	300
2-1	2334	14	387	0.99	2250	400
2-2	75586	44	789	6.05	3500	500
2-3	ND[c]	319	ND[c]	17	3500	700
3-1	67640	3382	655	72	5500	1200
3-2	40389	10097	467	201	7000	2500
3-3	ND[c]	18310	ND[c]	200	4000	3500
4-3	54922	23067	813	512	2500	2500
4-4	ND[c]	28668	ND[c]	749	2000	2000

[a]Autoclave cycle during 15 minutes at 120 °C.

[b]Needle of 27 gauge, 0.4 x 13 mm, 1 ml syringe.

[c] Not determined (ND) because the original hydrogel was very rigid and exceeded the measurement limit of the instrument.

Supplementary Table S5. Test article to negative control comparison.

Tester Strain	Fold over negative control-DMSO Test Article Extract[a]	Fold over negative control- Saline Test Article Extract[a]
TA98 without S9	1.1	1.3
TA98 with S9	0.9	1.2
TA100 without S9	1.0	1.0
TA100 with S9	1.0	1.0
TA1535 without S9	0.6	0.7
TA1535 with S9	0.6	0.9
TA1537 without S9	1.1	1.1
TA1537 with S9	1.3	1.4
WP2*uvrA* without S9	1.6	0.7
WP2*uvrA* with S9	1.0	0.8

[a]Value based on mean number of test extract revertants divided by the mean of negative control revertants. Values ≤1.0 represent no increase.

Supplementary Table S6. Positive to negative control comparison.

Tester Strain Positive Control	Fold over negative control DMSO Positive Controls[a]	Fold over negative control Saline Positive Controls[a]
TA98 without S9 + 2-nitrofluorene	49.5	50.4
TA98 with S9 + benzo[a] pyrene	42.4	46.7
TA100 without S9 + sodium azide	33.2	31.8
TA100 with S9 + 2-aminoanthracene	15.0	15.3
TA1535 without S9 + sodium azide	219.9	231.8
TA1535 with S9 + 2-aminoanthracene	10.1	17.1
TA1537 without S9 + ICR-191	5.4	6.0
TA1537 with S9 + 2aminoanthracene	7.1	8.2
WP2*uvrA* without S9 +methyl methasulfonate	27.9	17.0
WP2*uvrA* with S9 + 2-aminoanthracene	17.2	14.8

[a]Value based on mean number of positive control revertants divided by the mean of negative control revertants. Values ≤1.0 represent no increase.

Supplementary Table S7. Hematology parameters.

Group	Sex	Time	RBC (x106/µL)	HCT (%)	HGB (g/dL)	MCV (fL)	MCH (pg)	MCHC (g/dL)	WBC (x103/µL)	HET (x103/µL)	EOS (x103/µL)	BAS (x103/µL)	LYM (x103/µL)	MNO (x103/µL)
A	M	Basal	5.8 +/- 0.4	36.7 +/- 2.8	12.2 +/- 0.8	68.3 +/- 2.1	20.9 +/- 0.8	30.7 +/- 0.8	11.3 +/- 1.9	4.5 +/- 1.5	0.07 +/- 0.09	0.4 +/- 0.3	5.9 +/- 0.6	0.5 +/- 0.4
A	F	Basal	5.9 +/- 0.5	36.3 +/- 2.4	12.2 +/- 0.8	68.8 +/- 1.8	21.1 +/- 0.6	30.7 +/- 0.7	9.7 +/- 1.9	4 +/- 1.1	0.1 +/- 0.1	0.8 +/- 1.3	4.5 +/- 2.1	0.2 +/- 0.1
B	M	Basal	6 +/- 0.3	37.1 +/- 2.5	12.5 +/- 0.6	67.9 +/- 3.1	21 +/- 1.3	30.8 +/- 0.6	10 +/- 1.8	4 +/- 0.9	0.1 +/- 0.1	0.1 +/- 0.1	5.5 +/- 1.3	0.3 +/- 0.2
B	F	Basal	5.8 +/- 0.5	36 +/- 2.2	12 +/- 0.7	69 +/- 2	21.2 +/- 0.9	30.7 +/- 0.7	9.2 +/- 1.5	3.4 +/- 0.6	0.1 +/- 0.05	0.3 +/- 0.2	5 +/- 1.2	0.4 +/- 0.2
C	M	Basal	5.9 +/- 0.5	35.9 +/- 2.5	12.1 +/- 0.8	68.1 +/- 2	21 +/- 0.7	30.7 +/- 0.7	11.7 +/- 2.6	4.1 +/- 1	0.1 +/- 0.1	0.4 +/- 0.2	6.8 +/- 1.7	0.3 +/- 0.2
C	F	Basal	5.4 +/- 0.5	34.1 +/- 1.6	11.4 +/- 0.6	68.5 +/- 2.8	21.1 +/- 1.1	30.8 +/- 0.4	8.9 +/- 1.7	3.3 +/- 0.5	0.2 +/- 0.1	0.4 +/- 0.2	4.8 +/- 1.6	0.2 +/- 0.2
A	M	1m	6.1 +/- 0.3	38.2 +/- 2.1	12.5 +/- 0.5	68.3 +/- 2.4	20.5 +/- 0.9	30.1 +/- 0.5	9.9 +/- 2	3.8 +/-1	0.1 +/- 0.08	0.2 +/- 0.1	5.2 +/- 1.1	0.5 +/- 0.2
A	F	1m	5.7 +/- 0.2	33.6 +/- 1.3	11.5 +/- 0.6	67.3 +/- 1.6	20.5 +/- 0.6	30.5 +/- 0.5	9 +/- 1.9	3.6 +/- 1.5	0.1 +/- 0.1	0.4 +/- 0.3	4.1 +/- 0.8	0.6 +/- 0.3
B	M	1m	5.7 +/- 0.9	35.2 +/- 4.8	11.7 +/- 1.9	67.9 +/- 3.1	20.3 +/- 1	30 +/- 0.5	9.7 +/- 2.1	3.7 +/- 1	0.1 +/- 0.1	0.3 +/- 0.3	5.1 +/- 1.1	0.4 +/- 0.4
B	F	1m	5.7 +/- 0.4	33.8 +/-2.5	11.3 +/- 0.8	67.3 +/- 2	20.5 +/- 0.7	30.4 +/- 0.3	10.3 +/- 3.3	4.7 +/- 2.1	0.1 +/- 0.1	0.5 +/- 0.1	4.4 +/- 1.5	0.6 +/- 0.4
C	M	1m	6.2 +/- 0.5	39.5 +/-4.8	12.8 +/- 1.2	68.2 +/- 1.7	20.6 +/- 0.6	30.2 +/- 0.3	10.6 +/- 3	4 +/- 1.7	0.04 +/- 0.05	0.3 +/- 0.1	5.6 +/- 2.1	0.6 +/- 0.1
C	F	1m	5.7 +/- 0.2	34.6 +/- 3	11.4 +/- 0.8	67 +/- 2.2	20.3 +/- 0.7	30.3 +/- 0.6	9.8 +/- 2.2	3.2 +/- 0.9	0.2 +/- 0.1	0.4 +/- 0.2	5.5 +/- 2	0.4 +/- 0.2
A	M	3m	6.1 +/- 0.5	38.4 +/- 2.8	12.7 +/- 0.9	69 +/- 2.4	20.8 +/- 0.7	30.3 +/- 0.6	10.4 +/- 1.9	3.5 +/- 0.9	0.05 +/- 0.07	0.2 +/- 0.2	6.4 +/- 1.3	0.3 +/- 0.2
A	F	3m	5.5 +/- 0.3	34.6 +/- 1.6	11.2 +/- 0.5	67.2 +/- 1.9	19.6 +/- 0.7	29.2 +/- 0.4	8.1 +/- 1	2.9 +/- 0.6	0.09 +/- 0.09	0.4 +/- 0.2	4.1 +/- 0.7	0.5 +/- 0.2
B	M	3m	6 +/- 0.3	38.1 +/- 1.2	12.4 +/- 0.4	68.3 +/- 3.4	20.5 +/- 1.1	30.1 +/- 0.6	9.9 +/- 1.3	3.3 +/-0.6	0.1 +/- 0.1	0.2 +/- 0.1	5.3 +/- 1.3	0.3 +/- 0.2
B	F	3m	5.7 +/- 0.2	35.9 +/- 2.6	11.6 +/- 0.7	67.1 +/- 2.5	19.6 +/- 0.8	29.3 +/- 0.4	9 +/- 2.1	2.9 +/- 1	0.09 +/- 0.08	0.4 +/- 0.2	5 +/- 1.3	0.6 +/- 0.2
C	M	3m	6.4 +/- 0.6	40.8 +/-3.8	13.3 +/-1.4	68.4 +/- 2.3	20.6 +/- 0.8	30.1 +/- 0.3	9.8 +/- 1.6	3.4 +/- 0.8	0.05 +/- 0.06	0.2 +/- 0.3	5.8 +/- 1.3	0.4 +/- 0.2
C	F	3m	5.7 +/- 0.3	34.8 +/-1.2	11.3 +/- 0.5	66.6 +/- 3.2	19.9 +/- 1.1	29.8 +/- 0.4	9.7 +/- 2.4	2.9 +/- 0.7	0.1 +/- 0.1	0.4 +/- 0.3	5.9 +/- 2.6	0.3 +/- 0.1
A	M	6m	5.5 +/- 0.5	36.2 +/- 4.1	12 +/- 1.3	70 +/- 2.6	21.7 +/- 0.8	31.1 +/- 0.5	8.8 +/- 0.6	2.6 +/- 0.08	0.08+/- 0.09	0.5 +/- 0.2	5.1 +/- 0.9	0.5 +/- 0.3
A	F	6m	5.1 +/- 0.3	33.7 +/- 2	11 +/- 0.5	69 +/- 2.2	21.3 +/- 0.6	30.9 +/- 0.4	7.5 +/- 1.4	2.4 +/- 0.6	0.04 +/- 0.06	0.5 +/- 0.1	4 +/- 1.4	0.4 +/- 0.2
B	M	6m	5.7 +/- 0.2	37.2 +/- 2.3	12.4 +/- 0.7	68.9 +/- 2.2	21.6 +/- 0.8	31.4 +/- 0.5	7.6 +/- 1.1	1.9 +/- 0.4	0.09 +/- 0.08	0.4 +/- 0.2	4.8 +/- 1	0.5 +/- 0.2
B	F	6m	5.1 +/- 0.6	32.8 +/- 2.1	10.9 +/- 1	68 +/- 1.9	21.3 +/- 0.8	31.3 +/- 0.8	7.1 +/- 2.2	2.1 +/- 0.9	0.09 +/- 0.1	0.5 +/- 0.08	3.9 +/- 1.4	0.4 +/- 0.2
C	M	6m	6.6 +/- 2.1	43.6 +/- 11.1	14.5 +/- 4	70.6 +/- 1.9	22 +/- 0.9	31.1 +/- 0.7	9 +/- 2.2	2.6 +/- 0.7	0.06 +/- 0.1	0.3 +/- 0.1	5.2 +/- 1.7	0.7 +/- 0.4
C	F	6m	5.2 +/- 0.4	34 +/- 2	11.2 +/- 0.9	68.8 +/- 2.8	21.4 +/- 0.8	31.2 +/- 0.5	8.1 +/- 1.4	2.4 +/- 0.5	0.04 +/- 0.07	0.5 +/- 0.2	4.7 +/- 1.9	0.5 +/- 0.4
A	M	12m	5.7 +/- 0.3	33.3 +/- 3.1	11 +/- 0.8	68.3 +/- 2.8	20.3 +/- 1.1	29.7 +/- 0.6	9.9 +/- 0.5	4.1 +/- 0.9	0.03 +/- 0.06	0.7 +/- 0.2	4.5 +/- 0.3	0.5 +/- 0.05
A	F	12m	5.1 +/- 0.2	30.3 +/- 1.1	10.5 +/-0.6	69.6 +/- 1.8	20.4 +/- 0.4	29.3 +/- 1	7.8 +/- 1.6	3.2 +/- 1.2	0.2 +/- 0.08	0.5 +/- 0.2	3.6 +/- 0.7	0.3 +/- 0.1
B	M	12m	6.1 +/- 0.1	35 +/- 1.7	12 +/- 0.3	67.8 +/- 0.7	19.6 +/- 0.4	28.9 +/- 0.2	9.3 +/- 2.1	3.9 +/- 1.5	0.1 +/- 0.07	0.3 +/- 0.2	4.6 +/- 0.6	0.3 +/- 0.1
B	F	12m	5 +/- 0.5	30.7 +/- 1.5	9.8 +/- 0.9	67 +/- 0.5	19.6 +/- 0.3	29.3 +/- 0.2	8 +/- 1.8	2.3 +/- 0.7	0.1 +/- 0.1	0.8 +/- 0.4	4.4 +/- 1.4	0.3 +/- 0.1
C	M	12m	5.8 +/- 0.2	36.3 +/- 3.5	12.2 +/- 0.9	71.1 +/- 2.8	21.1 +/- 0.6	29.7 +/- 0.4	8.6 +/- 2	2.5 +/- 1.3	0.2 +/- 0.2	0.3 +/- 0.2	5.2 +/- 0.2	0.3 +/- 0.2
C	F	12m	5.4 +/- 0.4	32.3 +/- 3.2	10.7 +/- 1	67.7 +/- 2.3	19.8 +/- 0.6	29.3 +/- 0.1	9 +/- 1.3	2.9 +/- 0.4	0.1 +/- 0.03	0.6 +/- 0.03	5 +/- 0.9	0.3 +/- 0.1

RBC: red blood cells, HGB: hemoglobin, HCT: hematocrit, MCV: mean cell volume of red blood cells, MCH: mean cell hemoglobin, MCHC: mean corpuscular hemoglobin concentration, PLT: platelet, WBC: white blood cell, HET: heterophiles, EOS: eosinophils, BAS: basophils, LYM: lymphocyte, MNO: monocyte.

Data are presented as mean ± standard deviations. No statistically significant differences compared with vehicle control group at $P < 0.05$ were observed in relation to basal (analysis of variance, Dunnett post hoc test).

.

Supplementary Table S8. Serum chemistry data.

Group	Sex	Time	BUN (mg/dl)	CRE (mg/dl)	ALT (UI/l)	AST (UI/l)	TP (g/dl)	ALB (g/dl)	ALP (UI/l)
A	M	Basal	41.3 +/- 0.7	1 +/- 0.2	83 +/- 60	63 +/- 64	7.2 +/- 0.1	3.1 +/- 0.3	115 +/- 60
A	F	Basal	45.6 +/- 2	1 +/- 0.1	52 +/- 15	20 +/- 1	7.1 +/- 0.1	3.1 +/- 0.5	132 +/- 36
B	M	Basal	52.2 +/- 2	0.7 +/- 0.1	50 +/- 21	23 +/- 3	7.5 +/- 0.3	3.4 +/- 0.5	135 +/- 41
B	F	Basal	53.3 +/- 10.3	1 +/- 0.1	57 +/- 22	22 +/- 5	7.1 +/- 0.3	2.8 +/- 0.4	145 +/- 32
C	M	Basal	49.3 +/- 8.8	0.9 +/- 0.2	53 +/- 19	20 +/- 2	8.5 +/- 0.4	3.8 +/- 0.3	97 +/- 41
C	F	Basal	47.9 +/- 3	1 +/- 0.1	56 +/- 8	24 +/- 6	7.2 +/- 0.01	2.5 +/-0.02	147 +/- 8
A	M	1m	45.9 +/- 3.1	1 +/- 01	75 +/- 10	25 +/- 5	7.5 +/- 0.03	2.7 +/- 0.4	122 +/- 74
A	F	1m	40 +/- 4.2	1 +/- 0.2	80 +/- 11	22 +/- 6	7.5 +/- 0.2	2.6 +/- 0.2	121 +/- 27
B	M	1m	41 +/- 4	1 +/- 0.05	82 +/- 27	25 +/- 9	7.5 +/- 0.3	3.1 +/- 0.6	139 +/-59
B	F	1m	41.8 +/- 1.9	1 +/- 0.1	46 +/- 3	19 +/- 1	7.3 +/- 0.3	2.7 +/- 0.3	87 +/- 4
C	M	1m	42.2 +/- 9.8	1 +/- 0.2	66 +/- 22	24 +/- 4	7 +/- 0.2	2.4 +/- 0.2	92 +/- 38
C	F	1m	49.8 +/- 4	1.1 +/- 0.1	48 +/- 13	18 +/- 1	7.2 +/- 0.5	2.8 +/- 0.5	86 +/- 6
A	M	3m	45.6 +/- 3.7	0.7 +/- 0.2	64 +/- 18	21 +/- 4	6.2 +/- 0.2	2.6 +/-0.2	111 +/- 22
A	F	3m	49.3 +/- 4.8	1.1 +/- 0.2	52 +/- 11	17 +/- 1	6.1 +/- 0.4	2.9 +/- 0.4	111 +/- 24
B	M	3m	52.3 +/- 9.8	1.1 +/- 0.1	62 +/- 12	16 +/- 1	6.3 +/- 0.3	3.1 +/- 0.3	110 +/- 22
B	F	3m	53.7 +/- 7.9	1.1 +/- 0.2	51 +/- 10	19 +/- 6	6.8 +/- 0.4	3.1 +/- 0.2	98 +/- 16
C	M	3m	55.9 +/- 16	1 +/- 0.2	61 +/- 13	21 +/- 7	6.4 +/-0.1	3 +/- 0.1	99 +/- 9
C	F	3m	50.2 +/- 6.3	1.1 +/- 0.2	111 +/- 133	147 +/- 313	6.1 +/- 0.2	2.7 +/- 0.4	107 +/- 18
A	M	6m	42.4 +/- 7.3	1.6 +/- 0.05	40 +/- 6	11 +/- 2	5.6 +/- 0.2	2.8 +/- 0.3	92 +/- 15
A	F	6m	45.9 +/- 8.3	1.6 +/- 0.1	32 +/- 9	12 +/- 1	5.9 +/- 0.4	3 +/- 0.5	89 +/- 12
B	M	6m	33.8 +/- 6.8	1.3 +/- 0.1	36 +/- 12	15 +/- 5	5.7 +/- 0.5	2.7 +/- 0.4	89 +/- 12
B	F	6m	30.9 +/- 6.1	1.4 +/- 0.3	25 +/- 5	12 +/- 2	5.8 +/- 0.3	2.9 +/- 0.3	81 +/- 2
C	M	6m	45.4 +/- 20.8	1.6 +/- 0.1	29 +/- 9	11 +/- 1	5.6 +/- 0.3	2.6 +/- 0.2	105 +/- 31
C	F	6m	46.9 +/- 13.4	1.5 +/- 0.3	27 +/- 2	13 +/- 6	5.9 +/- 0.5	2.9 +/- 0.2	101 +/- 25
A	M	12m	35 +/- 4.5	1.1 +/- 0.1	35 +/- 7	19 +/- 10	6.1 +/- 0.3	3.3 +/- 0.5	99 +/- 25
A	F	12m	45.5 +/- 10.9	1.3 +/- 0.03	43 +/- 6	14 +/- 3	6.2 +/- 0.2	3.5 +/- 0.1	84 +/- 13
B	M	12m	24.8 +/- 3.5	1.2 +/- 0.1	70 +/- 4	17 +/- 3	6.1 +/- 0.1	3.4 +/- 0.1	81 +/- 12
B	F	12m	44.4 +/- 11.1	1.3 +/- 0.1	56 +/- 11	16 +/- 6	5.6 +/- 0.5	3.1 +/- 0.2	84 +/- 14
C	M	12m	29.1 +/- 5.7	1 +/- 0.1	51 +/- 10	15 +/- 4	6 +/- 0.1	3.3 +/- 0.4	86 +/- 54
C	F	12m	27.6 +/- 5.8	1.3 +/- 0.03	48 +/- 14	15 +/- 4	6.2 +/- 0.5	3.3 +/- 0.1	85 +/- 10

BUN: blood urea nitrogen, CRE: creatinine, ALT: alanine aminotransferase, AST: aspartate aminotransferase, TP: total protein, ALB: albumin, ALP: alkaline phosphatase.
Data are presented as mean ± standard deviations. No statistically significant differences compared with vehicle control group at $P < 0.05$ were observed in relation to basal (analysis of variance, Dunnett post hoc test).

Supplementary Figure S1. Chemical structure of chondroitin sulfate (CS) as chondroitin 4-sulfate (C-4S), Poliquaternium-10 (PQ10) and divinylsulfone (DVS).

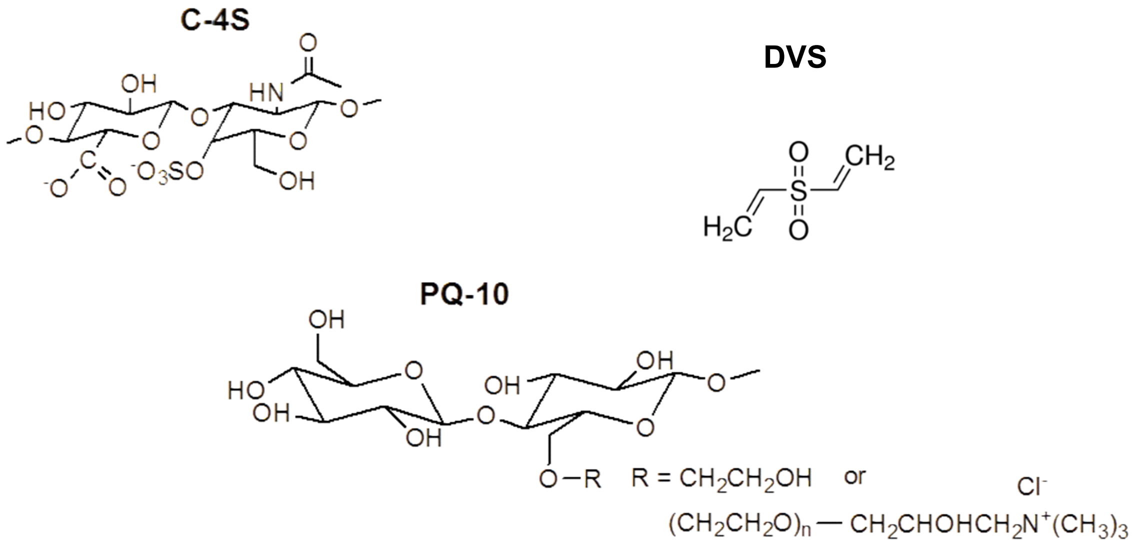

Supplementary Figure S2. Typical rheological profiles of G' (storage modulus), G'' (loss modulus), and η* (complex viscosity) for hydrogel PQ10 (3% w/v) / CS (0,3 % w/v) / DVS (3% w/w) (sample 3-3, Table S4) after heating process.

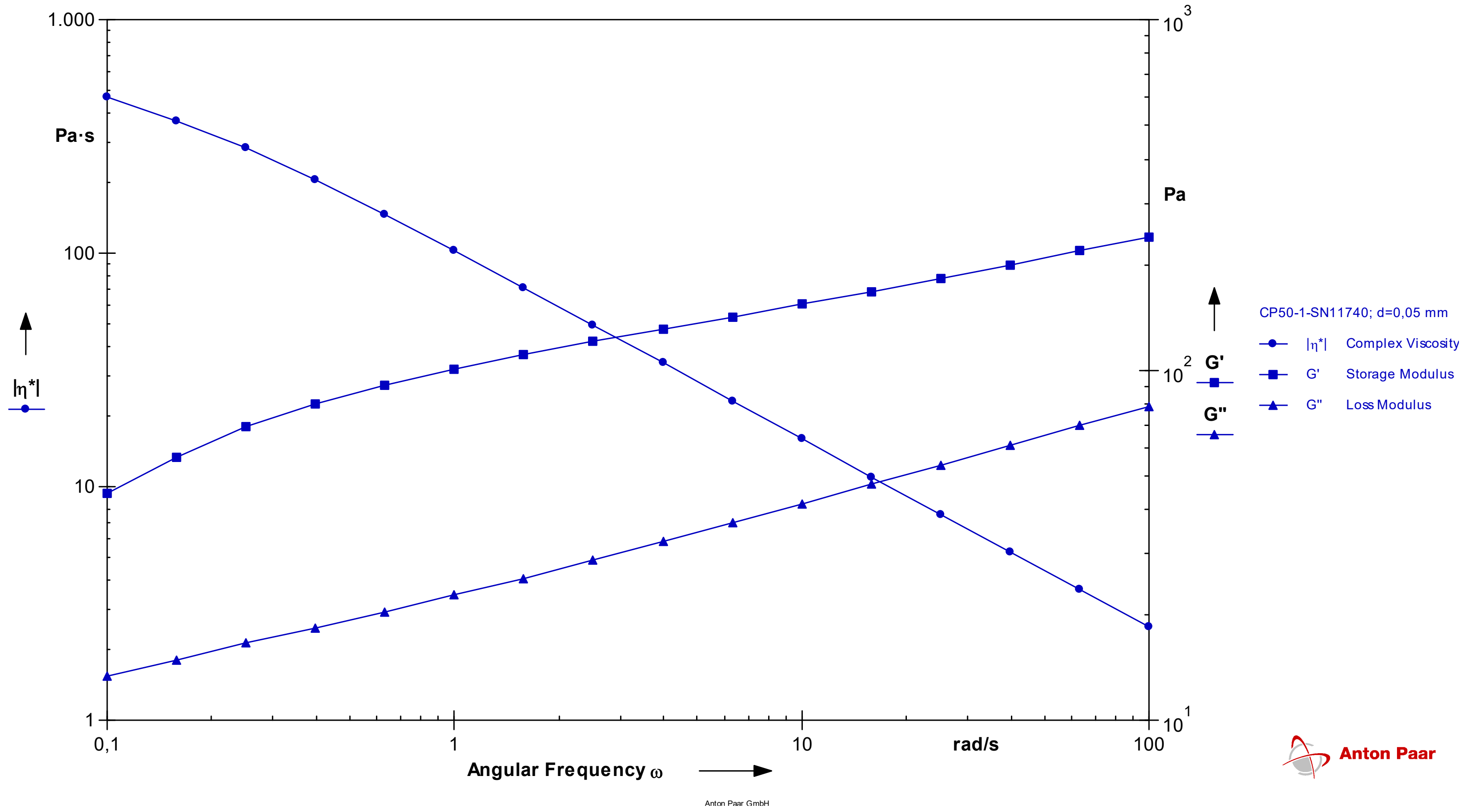

Supplementary Figure S3. Viscosity profile (η) vs Share Rate (γ) for hydrogel PQ10 (3% w/v) / CS (0,3 % w/v) / DVS (3% w/w) (sample 3-3, Table S4) after heating process.

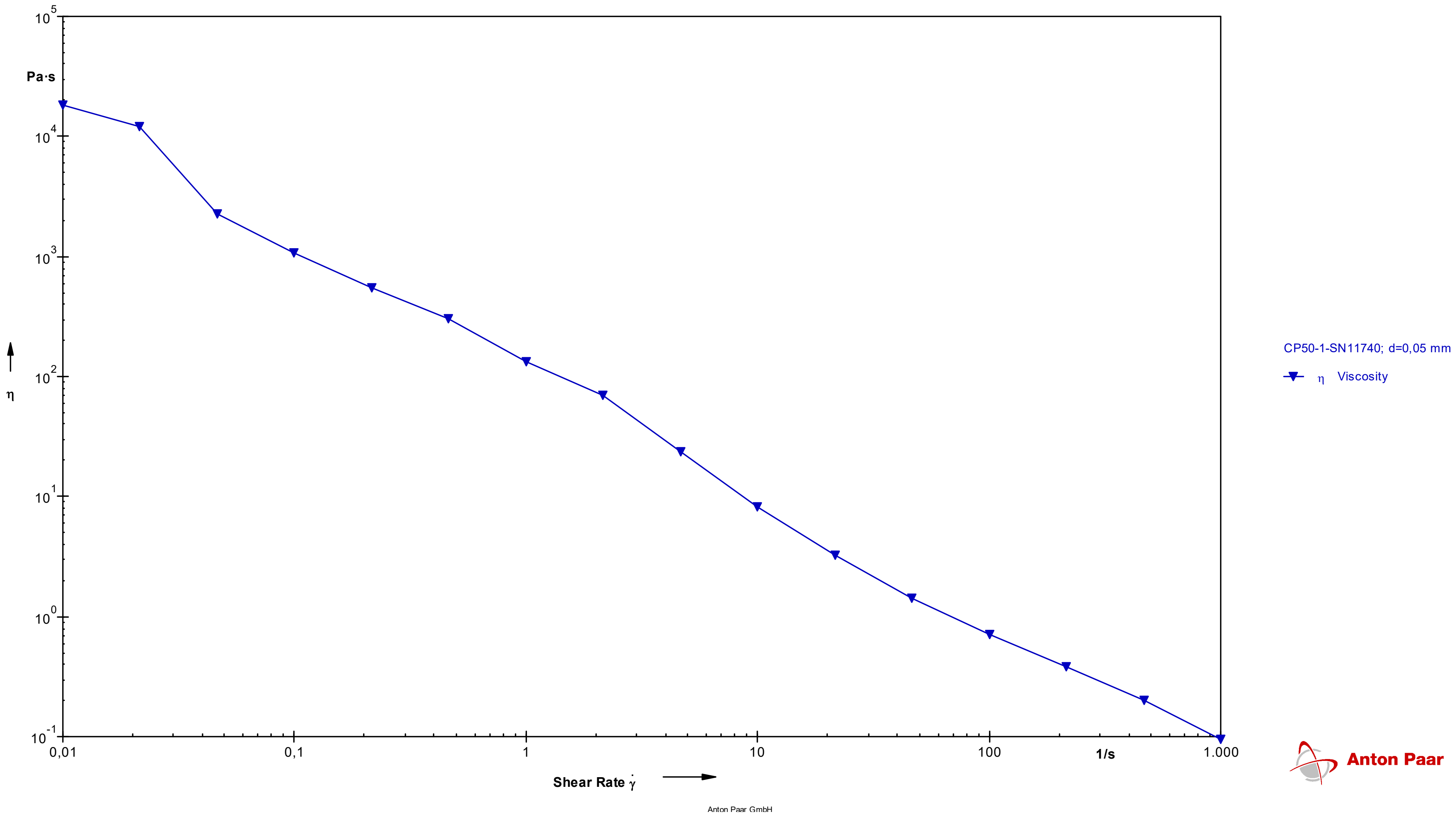

Figure S4. Representative radiological plates in rabbits from groups A, B, and C at different times 0, 6, and 12 months, respectively.

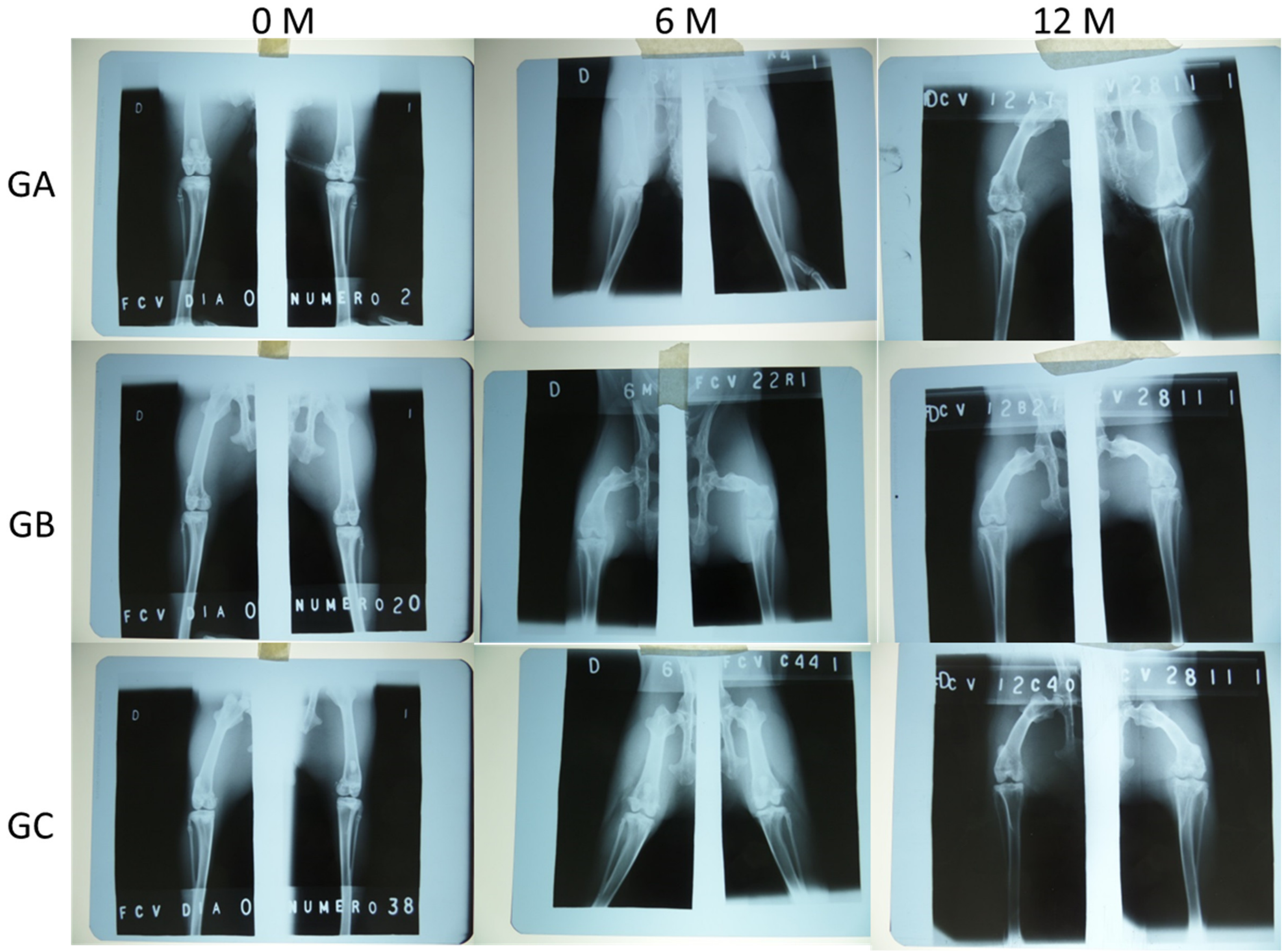

Supplement: Supplementary file 1 [file polymers-13-04426-s001.zip › polymers-1471359-supplementary.pdf]
